# Postnatal Development of Projections of the Postrhinal Cortex to the Entorhinal Cortex in the Rat

**DOI:** 10.1523/ENEURO.0057-22.2022

**Published:** 2022-06-27

**Authors:** Maria Jose Lagartos-Donate, Thanh Pierre Doan, Paulo J. B. Girão, Menno P. Witter

**Affiliations:** 1 Kavli Institute for Systems Neuroscience, Centre for Neural Computation, and Egil and Pauline Braathen and Fred Kavli Centre for Cortical Microcircuits, NTNU Norwegian University of Science and Technology, Trondheim 7491, Norway; 2 Department of Clinical Molecular Biology, University of Oslo and Akershus University Hospital, Lørenskog 1478, Norway; 3 Department of Neurology and Clinical Neurophysiology, St. Olav’s University Hospital, Trondheim 7030, Norway; 4 Department of Neuromedicine and Movement Science, NTNU, Trondheim N-7491, Norway

**Keywords:** context coding, functional development, memory, neuroanatomy, parahippocampal

## Abstract

The ability to encode and retrieve contextual information is an inherent feature of episodic memory that starts to develop during childhood. The postrhinal cortex (POR), an area of the parahippocampal region (PHR), has a crucial role in encoding object-space information and translating egocentric to allocentric representation of local space. The strong connectivity of POR with the adjacent entorhinal cortex (EC), and consequently the hippocampus, suggests that the development of these connections could support the postnatal development of contextual memory. Here, we report that POR projections of the rat develop progressively from the first to the third postnatal week starting in the medial EC (MEC) before spreading to the lateral EC (LEC). The increased spread and complexity of postrhinal axonal distributions is accompanied by an increased complexity of entorhinal dendritic trees and an increase of postrhinal-entorhinal synapses, which supports a gradual maturation in functional activity.

## Significance Statement

Postrhinal-entorhinal cortical interplay mediates important aspects of encoding and retrieval of contextual information that is important for episodic memory. To better understand the function of the postrhinal interactions with the entorhinal cortex (EC) we studied the postnatal development of the connection between the two cortical areas. Our study describes the postnatal development of the postrhinal-to-entorhinal projections as established with neuroanatomical and electrophysiological methods. The projections gradually reach functionally different areas of the EC, reaching the area involved in spatial functions first, followed by the part involved in representing information about objects and sequences of events.

## Introduction

Studies in humans and animals convincingly show that the parahippocampal region (PHR) in conjunction with the hippocampal formation (HF) play key roles in episodic memory by integrating information about “where,” “when” and “what” of experienced events (Arias et al., 2015; Bohbot et al., 2015; Heimer-McGinn et al., 2017; LaChance et al., 2019, 2022; Sugar and Moser, 2019; Sethumadhavan et al., 2020; LaFlamme et al., 2021). Since PHR is the major gateway to and from HF, a critical body of research has focused on addressing the functional contributions of each of the areas within PHR. Results indicated involvement of PHR in cognitive functioning (Ranganath and Ritchey, 2012; Eichenbaum, 2017) but also that its structure and functioning are altered in neurodegenerative and psychiatric disorders (Anderson et al., 2013; Roberts et al., 2016; Cheng et al., 2018).

The postrhinal cortex (POR) or its primate homolog, the parahippocampal cortex (PHC) is a main component of PHR, involved in the representations of the spatial layout of either familiar or novel objects, as well as features of, and patterns in the local context (Maguire et al., 1998; Burgess et al., 2001; Ranganath et al., 2004; Hayes et al., 2007; Furtak et al., 2012; Sethumadhavan et al., 2020). More recently, rodent studies emphasized that POR might be uniquely situated to translate egocentric information into an allocentric representation of local space. Whether this transformation happens in POR or by way of POR outputs interacting with neurons in other components of PHR, including the entorhinal cortex (EC) remains to be established (Gofman et al., 2019; LaChance et al., 2019, 2022).

One way to assess the relevant contributions of POR and the potential dependence on interactions with EC is to correlate the postnatal development of certain behavioral capacities with the development of associated connectivity patterns. Although the specific age points are still controversial, several studies in humans have shown that the ability to use contextual cues and create long-term object-in-context memories develop mainly from birth until late childhood (Mullally and Maguire, 2014; Lorsbach et al., 2015). Similarly, this seems to relate to changes in size of the parahippocampal place area during childhood (Lee et al., 2016; Meissner et al., 2019). Although rats are able to recognize and remember objects before postnatal day (P)17, contextual learning and memory for objects in location emerge later, between P17 and P31 (Westbrook et al., 2014; Ramsaran et al., 2016a, b).

In adult rats, POR provides inputs to both the medial EC (MEC) and lateral EC (LEC; Burwell and Amaral, 1998a; Burwell, 2000; Doan et al., 2019), but no data are available on the postnatal development of this input to EC. Spatially modulated neurons in MEC and the connectionally associated presubiculum and parasubiculum (PaS) emerge and fully develop at different postnatal time points. Head-direction cells in PHR have adult like properties at the end of the second postnatal week. In contrast the still not well-structured firing fields of grid cells present around P16, evolve gradually into adult-like functional firing properties between P16 and P34 (Langston et al., 2010; Wills et al., 2010). Very little to nothing is known about the development of functional neuron types in LEC.

In the adult animal, grid cells in MEC are sensitive to changes in nonmetric contextual cues, causing changes in grid cell periodicity (Pérez-Escobar et al., 2016). In view of the hypothesized interactions between POR and MEC in the formation of a stable allocentric representation of space in a specific context (Gofman et al., 2019; LaChance et al., 2019, 2022), we propose that the postnatal development of the projections of POR to MEC might impact the development of functional cell types in MEC. Since the system described above exhibits a gradual postnatal development of functionality, we aimed to assess the development of the POR-to-MEC projection by using retrograde and anterograde tracing, intracellular filling with neuronal quantitative evaluations, and *in vitro* voltage-sensitive-dye imaging from P2 to P23. We further compared the developmental timeline of POR projections to MEC and LEC.

## Materials and Methods

### Animals

A total of 110 female and male Long–Evans rats, aged between P2 and P23, were used in this study. Long–Evans pups were bred in-house. Breeding groups consisted of one female and one male housed in enriched cages containing toys and with free access to food and water. The cages were maintained on a 12/12 h reversed light/dark cycle. The males remained in the cage during pregnancy, parturition, and rearing of the pups. Cages were checked every morning and evening for pups, and the day on which pups were observed was considered P0. The day of perfusion was considered the age of the animal as reported in the results. To avoid unnecessary stress for the animals, the litter size was culled to ∼10 pups by P3. Pups were allowed to remain in the nest with the mother until weaning at P21. All procedures followed locally approved protocols that adhere to the European Communities Council Directive and the Norwegian Experiments on Animals Act and the local directives of the responsible veterinarian at the Norwegian University of Science and Technology.

### Surgeries

All surgeries were conducted under isoflurane anesthesia. Animals were placed in an induction chamber and anesthetized until they could be moved to a stereotaxic frame. Neonatal animals (P2–P13) were held under anesthesia with a neonatal mask (model 973-B; Kopf), with the palate bar width reduced to fit the inside of the mouth better, and the head was fixed with zygoma ear cups (model 921; Kopf). Older animals (P14–adult) were kept under anesthesia with a small adult mask and head fixed with blunted ear bars.

Before incision, the skin was disinfected with iodine, and a local analgesic, bupivacain (0.2 ml/100 g bodyweight of a 0.5 mg/ml solution; Marcain, Astra Zeneca), was injected subcutaneously at the place of incision. The incision was made with a small-sized and sharp tipped scissor. Afterwards, the mouthpiece and ear cups were adjusted so that bregma and λ were aligned horizontally. Before injecting, two holes were drilled in the bone, one over the planned place of injection and another over the posterior extreme of the sagittal sinus. The place of injection was measured using the junction of the transverse sinus as a reference for the anteroposterior coordinate, the sagittal sinus as a reference for the mediolateral coordinate and the level of the dura as a reference for the dorsoventral coordinate. For the injection, glass capillaries with an outer diameter of 20–25 μm (30-0044, Harvard Apparatus; pulled with a PP-830 puller, Narishige) were used to make the glass micropipette. Biotinylated dextran amine (BDA; 5% in phosphate buffer (PB; 0.125 m in H_2_O; pH 7.4), 10 kDa, D1956, Invitrogen] was iontophoretically injected through the micropipettes, filled with 0,5 μl of the BDA solution into POR (6–7 μA, alternating currents, 6 s on/6 s off, for 5–15 min, 51595; Stoelting) for anterograde tracing. Since the size and shape of the brain changes during postnatal development, the coordinates to target POR were different between the youngest and oldest rats (AP: ∼0.5 mm from the posterior transverse sinus as measured 4 mm lateral to the midsagittal sinus, ML: 3–4.5 mm from midsagittal sinus, depth: ∼1–1.5 mm, respectively). Based on the location of the center of the injection site, the injections were subsequently classified as vPOR or dPOR. For retrograde tracing, fast-blue (0,3 μl, FB; 1% in 0.125 m PB, EMS Chemie) or retrobead green (0.3 μl, Lumafluor) were mechanically injected through micropipettes into MEC. For this objective we used the following coordinates: AP: ∼0.5 mm from the posterior transverse sinus, ML: 3–4.5 mm, depth: ∼2–3 mm. During surgery, appropriate amount of sterile saline (room temperature) was administered subcutaneously to avoid dehydration. Rat pups were also administered Rimadyl during surgery as a postsurgery analgesic (1 ml/100 g bodyweight of a 0.5 mg/ml solution; Rimadyl, Pfizer). After the injections were finished, the incision was sutured, and the pups were allowed to recover under a heating lamp. When fully awake, the animals were returned to maternal care until the time of sacrifice.

### Perfusion and tissue processing

We killed the animals 18–30 h after surgery under terminal anesthesia with isoflurane. The thorax was opened and cold Ringer’s solution (8.5 g NaCl, 0.25 g KCl, and 0.2 g NaHCO_3_ per liter of H_2_O, pH 6.9) was transcardially perfused through the body. When the liver turned pale the perfusion solution was changed to a 4% solution of freshly depolymerized paraformaldehyde (PFA; catalog #140; Merck) in PB (pH 7.4) until the body was sufficiently stiff. The brains were extracted and postfixed overnight at 4°C in PFA before being moved to a cryoprotective solution (20% glycerol, 2% dimethylsulfoxide in 125 mm PB, pH 7.4).

Fifty-micrometer sagittal sections were cut on a freezing microtome (HM-430 Thermo Scientific) and sections were collected in five equally spaced series. The first series was directly mounted on Superfrost Plus slides (10149870, Thermo Scientific) and dried overnight on a warming plate at 30°C for subsequent Nissl staining.

The other series were placed in the cryoprotecting solution and stored at −22°C until further usage. The mounted series for Nissl staining were dehydrated in increasing ethanol solutions (50%, 70%, 80%, 90%, 100%, 100%, 100%) followed by 2 min in xylene (VWR) to clear the sections. After that, the sections were rehydrated in decreasing ethanol solutions (opposite order as the dehydration protocol) and placed in cresyl violet solution for two to 6 min. Subsequently, the sections were rinsed in water and placed in 50% ethanol containing acetic acid to differentiate the staining. Finally, the sections were dehydrated in ethanol, cleared in xylene and coverslipped with Entellan (107961, Merck).

For delineation purposes, adjacent series were immunostained for calcium-binding proteins parvalbumin (PV), calretinin (CR), and calbindin D-28 (CB) with primary antibodies (mouse anti-PV, 1:1000, Sigma-Aldrich Merck Millipore; mouse anti-CR,1:2000, Millipore; rabbit anti-CB, 1:3000, Swant).

All sections were rinsed three times for 10 min in PB (125 nm PB, pH 7.4) and preincubated for 1.5 h in 5% normal goat serum in TBS-TX (solution of 50 mm Tris, 0.87% sodium chloride and 0.5% Triton X-100). Next, individual series of sections were incubated with primary antibodies in TBS-TX overnight at 4°C. After rinsing, sections were incubated in a secondary goat anti-mouse antibody coupled to biotin (Sigma-Aldrich B7151, 1:100 in TBS-TX for 90 min at room temperature), followed by incubation with the Vector ABC kit according to the manufacturer’s specifications (Vector Laboratories Inc., Peroxidase standard PK-Vectastain ABC kit 400), rinsed in TBS, incubated with DAB for 1–10 min and rinsed in Tris/HCl solution to stop the reaction. Then sections were mounted on glass slides from a 0.2% gelatin solution, and dried. After 2 d, the sections were dehydrated through increasing concentrations of ethanol to xylene and coverslipped with Entellan (Merck).

### Immunohistochemistry for BDA anterograde tracer visualization

To visualize BDA, free-floating sections were washed in 125 mm PB three times for 10 min each. Endogenous peroxidases were blocked in three washes with 3% hydrogen peroxide in PB for 10 min each, followed by three washes in PB for 10 min each. The sections were subsequently washed in TBS-TX (50 mm Tris, 150 mm NaCl, 0.5% Triton X-100, pH 8.0) three times for 10 min each, followed by a 90-min incubation in TBS-TX with Alexa-conjugated streptavidin (Alexa Fluor 546, S11225, Invitrogen) in a 1:200 solution in TBS-TX overnight at 4°C. Then the sections were rinsed three times for 5 min in Tris-HCl and mounted on glass slides, air-dried overnight, cleared in toluene, and coverslipped with Entellan (Merck Darmstadt, catalog #107961). In some cases, Nissl staining was necessary to aid the delineation of brain regions.

To obtain digital images of all experimental material, we used automated slide scanners equipped with the appropriate brightfield and fluorescent imaging systems (Zeiss Axio Imager M1/2 and Zeiss Mirax Midi). We used 20× objectives (NA 0.8) and all files were postprocessed with the use of Adobe photoshop and illustrator (CS6, Adobe Systems).

### Quantitative assessment of the POR injections and the distribution of the projections

After the sections were digitized and qualitatively studied, we selected those POR anterograde injections where the center of mass was clearly in POR; this resulted in 51 representative injections being included. We aimed to obtain, for each of these experiments, a realistic estimation of the location of the injection in POR and the location of the anterogradely labeled axons in MEC. To achieve this, we first plotted the center of mass for each injection site into the 3D Waxholm space atlas reference frame (Papp et al., 2014).

**Figure 1.**
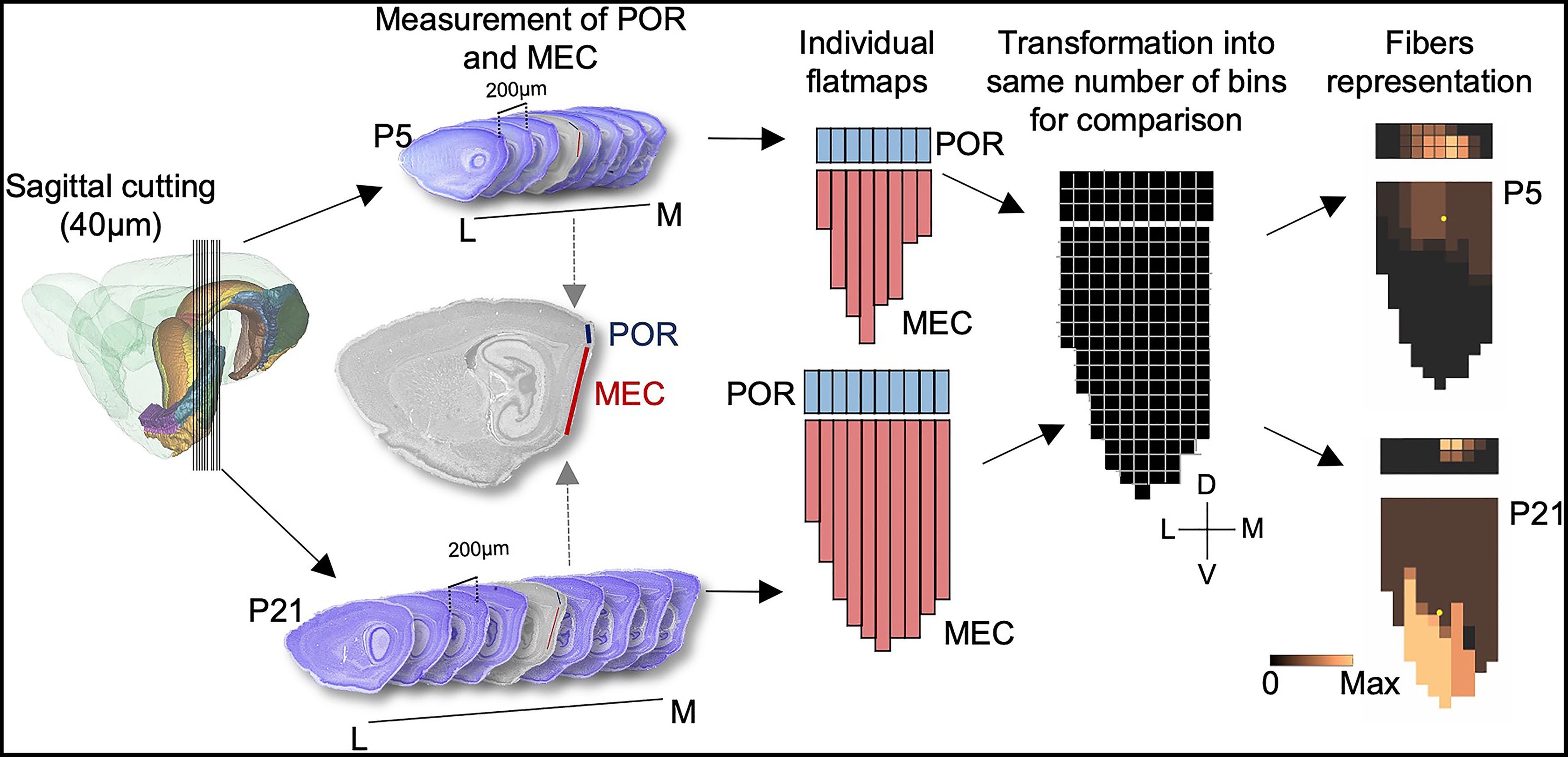
Methodology used to create flatmaps. The rat brains were cut in five series of 40-μm-thick sagittal sections. One series was consecutively mounted and used for Nissl staining. Flatmaps were subsequently created for quantitative assessment of POR injections and axonal distribution by measuring the dorsoventral extent of POR and MEC in each Nissl section (example: red line, in MEC, and blue line, POR) and creating individual flatmaps for each of the MEC and POR regions. All measurements were aligned along a horizontal line representing the border between MEC and POR. To compare the different flatmaps with each other, we normalized individual flatmaps to the same number of equally sized bins. At last, the number associated with each projection density pattern (i.e., 1–4) was entered in each bin. D: dorsal; V: ventral; M: medial; L: lateral.

Second, we produced individual flatmaps of each brain by measuring the dorsoventral length of POR and MEC in each sagittal section and plotted the distance between each section, including the section thickness. These measurements were projected into a 2D flatmap representation (Fig. 1). The border between MEC and POR in the sagittal sections was used to align all sections. We subsequently measured the position, and extent of labeled POR axons along the dorsoventral axis of MEC (by using Panoramic Viewer software, 3DHistech). The data were stored in excel (Microsoft) and were further processed using a locally written MATLAB script (R2015b, MathWorks). Since brains of animals of different ages have different sizes, we converted the absolute measurements obtained for each individual flatmap into normalized values. For this, we binned the MEC and POR along their dorsoventral and mediolateral axes in each individual flatmap into equally sized bins. This methodology has been described in more detail previously (Sugar and Witter, 2016).

Third, in all sections, we subsequently represented the density of fibers in each bin by a numerical value ranging between zero (no fibers) to four (very dense plexus). We did not distinguish between superficial and deep layers of MEC. These data were subsequently entered onto the normalized flatmaps described above. A similar procedure was used to represent the coverage of the injection site, where a value of “4” represented the center of the injection and values from 3 to 1, represent areas at an increasing distance from the center of mass of the injection, which showed decreasing intensity of labeling, respectively.

In order to quantitatively assess the effect of age and position of the injection site in POR on the distribution of the projection in MEC, four different statistical tools were used. In all statistical tools, we used age of subjects and center of mass of the injection site in POR as the independent variables. The coordinates for center of mass of the projection in MEC, along both the dorsoventral and the mediolateral axis, were used as the dependent variable. These values were tested for normality of distribution by both visual inspection of the plotted groups, and subsequent use of the Anderson–Darling test. In all analyses, the age of the subjects was pooled into three discrete ordinal categories, namely first, second and third postnatal weeks. In three of the statistical tests, namely k-means cluster analysis and one-way and two-way ANOVA tests, the position of injection site in POR was pooled into two nominal categories, ventral and dorsal (vPOR and dPOR), based on Nissl and immunostaining (see above and as described in Result, Nomenclature). To avoid subjective bias, we used a fourth statistical test, the Pearson’s correlation coefficient analysis. In this test, discrete values of position of injection site in the POR were used as the independent variable. These values were calculated by plotting the approximate individual centers of the injection points in the 3D Waxholm space atlas reference frame (Papp et al., 2014), and subsequently calculating its normalized position in the frame by using an affine transformation of the individual 2D sections extracted along the anterior-to-posterior axis of the model POR. This method was chosen to take into account the ontogenetic stage and individual variations between the subjects, in an attempt to use a reference frame that permits comparability. K-means cluster analysis and one-way ANOVA tests were conducted in SPSS, while Anderson–Darling normality test, two-way ANOVA test and Pearson’s correlation analysis were conducted in MATLAB.

### Immunohistochemistry for bassoon, synaptophysin, and CB

Sections from retrograde and anterograde tracing experiments were assessed for the distribution of CB or bassoon and synaptophysin using immunohistochemistry. Free-floating sections were washed in 125 mm PB three times for 10 min each. Endogenous peroxidases were blocked in three washes with 3% hydrogen peroxide in PB for 10 min each, and then washed again in PB three times for 10 min each. The sections were subsequently washed in TBS-TX (50 mm Tris, 150 mm NaCl, 0.5% Triton X-100, pH 8.0) three times for 10 min each, and subsequently incubated in a 1:500 solution with TBS-TX and primary antibodies for bassoon and synaptophysin (Syn; mouse anti-Bassoon Abcam; rabbit anti-Syn Abcam) or CB (rabbit anti-CB, Swant) overnight at 4°C. The day after, sections were rinsed three times for 10 min in TBS-TX and incubated for 90 min in a 1:1000 solution of secondary antibodies (Alexa Fluor 488 or 546, Invitrogen) in TBS-TX. Afterwards, sections were rinsed three times for 5 min in Tris-HCl and mounted on glass slides, dried overnight, cleared in toluene, and coverslipped with entellan (Merck Darmstadt, catalog #107961).

### Intracellular injections

An additional series of experiments was done in rat pups between 11 and 21 weeks old to identify neurons in MEC that might receive synaptic inputs from POR. To this end, we injected the anterograde tracer BDA into POR (same procedure as described above). After 7–8 d survival time, the rat pups were anesthetized and perfused as previously described. Hereafter, the brain was extracted and postfixed by 4% PFA at 4°C for 4 h and change into 0.125 m PB solution. The day after, the tissue was cut in 400-μm-thick sagittal sections with the use of a vibratome (Leica, model VT 1000S; bath fluid 0.125 m PB).

We selected the slices where MEC LII was clearly visible and used these for the intracellular filling of cell bodies in Layer II of the MEC. A total of 11 neurons, from 11 different rat pups, were intracellularly injected with Alexa Fluor 568 hydrazide dye (A10441, Thermo Fisher). The sections were rinsed in 0.125 m PBS and then mounted in a chamber filled with PBS on a fixed-stage upright microscope (Axio Examiner; Zeiss), which was equipped with a PlanApo 20×, 1.0 NA water dipping objective. Sections were imaged using IR-DIC illumination, resolving neuronal details. We used a micromanipulator (Luigs & Neumann) to impale single neurons with a 70- to 130-MΩ glass pipette (Harvard Apparatus; microelectrode-tip diameter 0.5–0.8 μm) filled with 10 mm Alexa Fluor 568 hydrazide dye in 200 mm KCl (Merck) solution. With a custom-made iontophoretic injection device, randomly selected MEC Layer II neurons located dorsally in MEC, were intracellularly filled. For at least 10 min, a negative current was administered to the glass pipette (2 nA, 500 ms on, 500 ms off) to entirely fill the impaled neuron. This implied that fine details, for instance dendritic spines and fine distal branches of the dendrites furthest away from the soma could be clearly seen. Once the filling was completed, the sections were immediately transferred and postfixed overnight in PFA at 4°C. Hereafter, immunohistochemistry to visualize the BDA anterograde tracer was performed.

### 3D reconstruction and analysis of neurons

Following protocols published in detail previously (Kononenko and Witter, 2012; Czajkowski et al., 2013), image stacks from the intracellularly filled neurons were obtained using a confocal microscope (Zeiss LSM 880 AxioImager Z2). Image files were converted with AMIRA 5.3.3 software (TGS, Mercury Computer Systems). The intracellularly filled neurons from MEC LII were reconstructed by using an extended custom developed Amira plug-in, Skeleton Tool. We measured the total dendritic length, arborization of dendritic trees, number of spines per dendritic branch order, spine density and the proximity of spines to the soma. Furthermore, a Sholl analysis was performed (Sholl, 1953), which demonstrates the number of branches crossing concentric spheres of increasing radius (Δr = 20 μm) around the soma.

**Figure 2.**
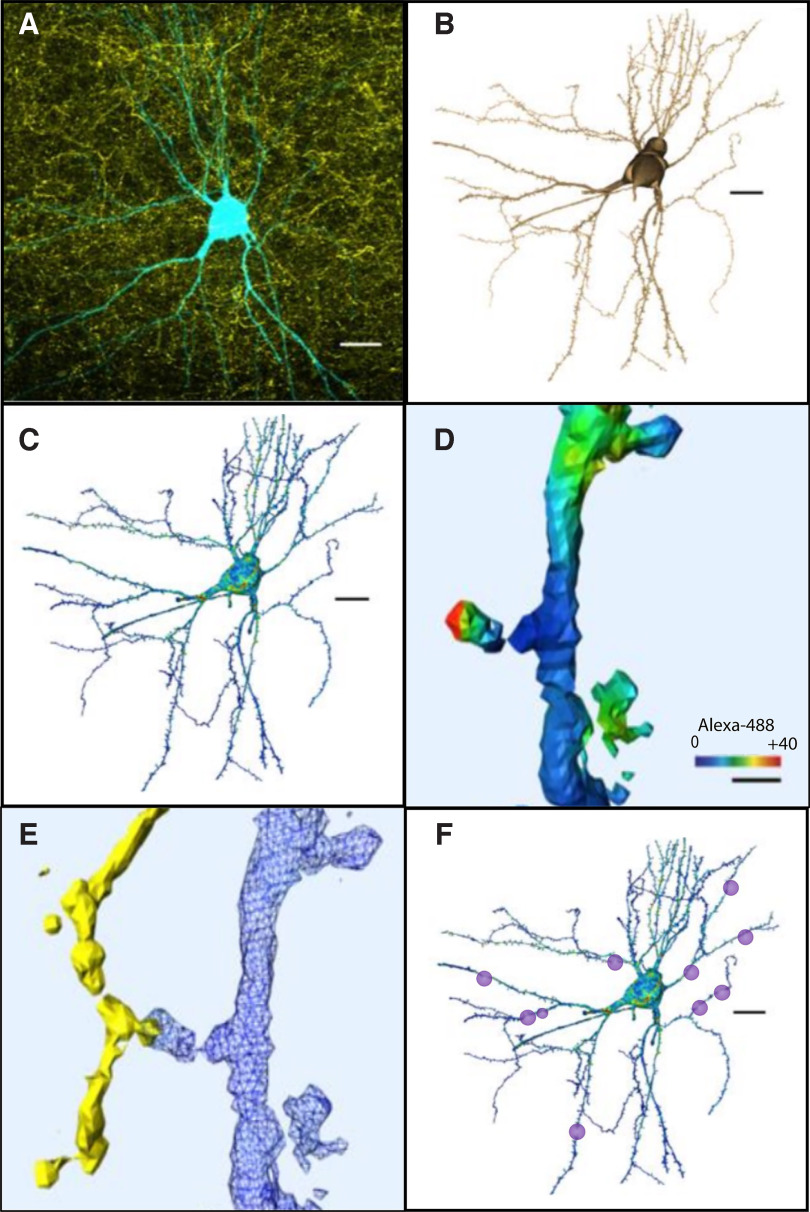
Reconstruction process from confocal imaging for identification and quantification of potential synaptic contacts. ***A***, Confocal image showing POR axons labeled with BDA (yellow) and an intracellularly filled MEC neuron (AF568, cyan). ***B***, Reconstruction with AMIRA of the dye injected MEC neuron. ***C***, Surface rendering and POR input mapping on the 3D-reconstructed neuron. ***D***, ***E***, Surface rendering of the dendritic fragment with POR input mapping. Average fluorescence intensity of BDA fibers is mapped and color coded (red color means the intensity is high and close enough to the dentridic spine that is considered putative synapse). ***F***, The putative synaptic contacts are marked and quantified. Scale bars: 20 μm (***A***–***C***, ***F***) and 1 μm (***D***).

The presence of putative synapses was assessed by considering BDA-positive voxels that were within 300 nm from the MEC LII dendritic surface. The proximity of these voxels was indicated as a heatmap by the software (Fig. 2). For each such potential overlap, the image was analyzed, according to the criteria for a presynaptic bouton: the diameter of the axonal swelling must be three times bigger than the preceding fiber (Wouterlood et al., 2008). This analysis revealed the number, and location of putative synapses and their location on shafts versus spines, proximity to the soma and dendritic branch order.

### Slice preparation for electrophysiology

Slices (400-μm thickness) were prepared from Long–Evans pup rats among 3 and 23 d of age. We used 48 animals in total. Animals were anesthetized with isoflurane (Isofane, Vericore), subsequently decapitated, the brain quickly removed from the skull and placed in oxygenated (95% O_2_–5% CO_2_) ice-cold ACSF (in mm; 124 NaCl, 5 KCl, 1.25 NaH_2_PO_4_, 2 MgSO_4_, 2 CaCl_2_, 10 glucose, 22 NaHCO_3_). To maintain the connectivity between POR and MEC, tilted sagittal slices were cut with an angle of ∼5° with the midsagittal plane and 85° with the dorsal surface of the hemisphere (Koganezawa et al., 2015). Slices were transferred onto a fine-mesh membrane filter (Omni pore membrane filter, JHWP01300, Millipore) held in place by a thin Plexiglas ring (11-mm inner diameter; 15-mm outer diameter; 1- to 2-mm thickness), maintained in a moist interface chamber, containing ACSF, and continuously supplied with a mixture of 95% O_2_ and 5% CO_2_ gas, which was moistened by leading the gas through ACSF before it entered the chamber. The ACSF was maintained a t 32°C. For all experiments, slices rested for at least 1 h until used one by one in the recording chamber perfused with ACSF.

### Electrophysiology: voltage-sensitive dye (VSD) imaging

Each slice was placed in the recording chamber where the slices were perfused with ACSF and positioned under a fluorescence microscope (Axio Examiner, Zeiss). In the chamber the slice was stained for 3 min with the VSD RH-795 (R649, Invitrogen, 0.5% in ACSF; Koganezawa et al., 2015). Slices were excited with 535 ± 25-nm light (bandpass), reflected by a dichroic mirror (half reflectance wavelength of 580 nm). Epifluorescence was detected through a long-wavelength pass filter (50% transmittance at 590 nm) with a CMOS-camera (MiCAM Ultima, BrainVision; 100 × 100 pixels array). When the optical recording was triggered, an electronically controlled shutter built into the light source (HL-151, Brain Vision) was opened 500 ms before the start of recording to avoid both mechanical disturbances caused by the shutter system and unnecessary bleaching of the dye. Subsequently, the optical baseline was allowed to stabilize for 50 ms before stimuli were delivered. For all experiments, 512 frames at a rate of 1.0 ms/frame were acquired. To represent the spread of neural activity, we superimposed color-coded optical signals on the bright-field image. In this procedure, we applied a color code to the fraction of the optical signal, which exceeded the baseline noise. To reduce baseline noise, we averaged eight identical recordings acquired with a 3-s interval directly in the frame memory. Optical signals were analyzed off-line using BrainVision analysis software. Changes in membrane potential were evaluated in a ROI as fractional changes of fluorescence (ΔF/F). The stimulation electrode was a tungsten bipolar electrode with a tip separation of 150–200 μm. We used 261 slices, and in some, several stimulation positions were tested subsequently. In all experiments, a single pulse stimulation (300 μs, 0.1–0.3 mA) was used for each stimulation cycle.

In one set of experiments, a low dose of the GABA_A_ antagonist bicuculline (5 μm bicuculline methiodide; 14343; Sigma-Aldrich) was used to assess the activity of GABA system during the first three weeks of postnatal development. After single pulse stimulation, the slices were perfused with GABA_A_ antagonist bicuculline for 10 min. Then, an identical single pulse stimulation was applied, and the effects were measured. To compare the effect of this GABA blocker, we measured and compared the total area under the curve that was above baseline.

To identify the position of stimulation and recording in the VSD experiments, slices were postfixed in 4% PFA for up to one week and subsequently kept in PBS with 30% sucrose for >10 h and cut at 50-μm thickness using a freezing microtome. Mounted sections were Nissl-stained with cresyl violet and coverslipped using Entellan (see above). Digital images of sections were combined with the optical imaging data to identify the region in which changes in neural firing occurred.

We measured the activity after beginning of the stimulus artifact in superficial and deep layers of POR and in superficial and deep layers of MEC by using BrainVision analyses software. To evaluate the latency and the strength of the signal in POR and MEC, we took eight equidistant measurements from the beginning of the stimulus artifact (in POR) into MEC (see Results for further details). To assess the latency, we measured the time it took for the signal to spread from one equidistant measurement point to the following equidistant measurement point. To assess the strength, we measured the maximum value of the peak at each equidistant measurement point. Statistical analysis between were taken 13 s after stimulation. Bicuculline versus no-bicuculine data were subjected to repeated measures ANOVA and, *post hoc* tests were performed (Tukey’s HSD test). Differences were considered statistically significant when *p* < 0.05.

## Results

### Nomenclature

We first needed to delineate the borders of the POR with the surrounding areas from the first until the fourth week of postnatal development. For this we used and adapted criteria, previously described in the adult rat. These are based on combinatorial analyses of patterns of PV, CB, and CR immunohistochemistry and Nissl staining (Boccara et al., 2015; Doan et al., 2019). In adult rats, POR is positioned caudal to perirhinal cortex, ventral to the visual cortex, and mostly dorsal to the rhinal fissure where it borders EC. This overall cortical arrangement can be recognized from birth onward, though the position of all areas is more dorsal on the cortical surface than in the adult. During the first weeks of postnatal development these cortical regions gradually move toward a more ventral position in the brain, which is clearly illustrated by the position of the rhinal sulcus.

POR can be divided into two different cytoarchitectonic subfields, a ventral (vPOR) and a dorsal part (dPOR; Burwell, 2001). In adults, vPOR is defined by the presence of ectopic Layer II cells near the border with the EC and vPOR is dysgranular along the rostro-caudal axis. Moreover, vPOR Layers II and III have small neurons in contrast to the ventrally adjacent MEC, which has larger neurons in Layer II. Furthermore, the CB-immunoreactivity is stronger in vPOR than in dPOR and MEC. That difference is more evident between Layer III of dPOR and vPOR. Contrary to EC, which has clear neuropil immunoreactive for CB in Layer I, POR lacks this CB positive labeling in Layer I. Additionally, MEC shows a strongly PV-positive neuropil staining which is completely absent in vPOR. CR staining further helps to distinguish between EC and POR. In EC the immunoreactivity for CR is light, whereas in POR it is very strong, which is similar to the rest of the neocortex. It is however possible to distinguish the border of POR from the surrounding neocortex since the immunoreactivity for CR in POR is stronger than the neighboring cortical areas.

**Figure 3.**
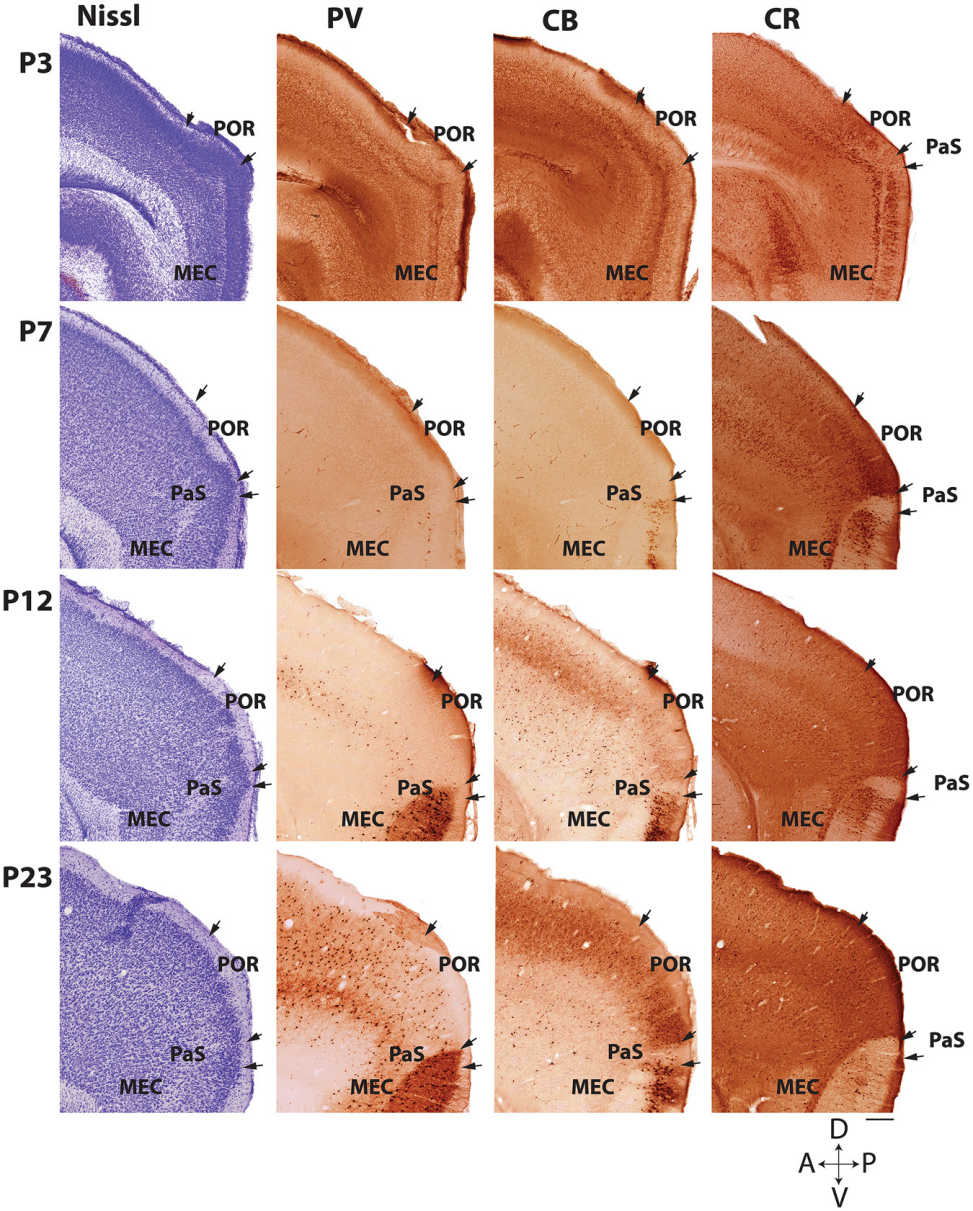
Defining POR in sagittal sections at different postnatal ages. Sagittal sections showing the POR and adjacent regions (PaS; MEC) at postnatal ages (P): P3, P7, P12, and P23. Adjacent sections stained for Nissl (Nissl), parvalbumin (PV), calbindin (CB), and calretinin (CR) are shown from left to right, respectively. The combination of Nissl staining and the immunohistochemistry for PV, CB, and CR allows accurate delineation of the borders between POR and the adjacent regions. D: dorsal; V: ventral; A: anterior; P: posterior. Scale bar: 100 μm.

Following these criteria, the boundaries of POR with its surrounding areas and the border between vPOR and dPOR were quite clear during the second and the third week of postnatal development (Fig. 3). However, in the first week of postnatal development, these criteria were less trivial to apply since neurons and neuropil do not yet stain for PV and CB in POR nor MEC. This is in line with previous reports that PV expression in the cortex only starts around P8–P10 (Du et al., 1996; Goldberg et al., 2011) and that CB expression shows a similar delayed expression (Ray and Brecht, 2016). In addition, neurons, and cortical lamination were not fully mature (Canto et al., 2019; Donato et al., 2015) until the end of the second week, and in line with this, also Nissl-based criteria were not easily applicable in the first postnatal week. At these early ages (P3–P7; Fig. 3), the positive CR immunoreactivity in Layer V of EC marked the border between EC and POR, and we identified the border between POR and the rest of neocortex, based on the difference in laminar structure, which was more regular and evident in the neocortex than in POR (Fig. 3).

It is well established that in the adult rat the position of the PaS is quite variable and may extend laterally and becomes inserted in between MEC and vPOR. This can be easily missed, particularly in young postnatal animals. However, in animals older than P8 the cytochemical differences between PaS, MEC, and vPOR were clear. Such a lateral extension and insertion of PaS could be seen as a small though prominent cluster of neurons with approximately the same size of MEC Layer II, although lacking a marked Layer II/III differentiation. The PaS showed a lack of reactivity for CB, which contrasted with the moderate to strong reactivity to that protein of the deep half of Layer II in MEC and vPOR (Fig. 3; Boccara et al., 2015). Likewise, the CR distribution in the dorsal part of MEC showed a marked densely labeled cluster of neurons in Layer III, whereas the superficial component of PaS was almost devoid of CR-positive neurons. This lack of CR staining also differentiated PaS from vPOR since the overall neuropil staining was much denser in vPOR than in PaS, even in the youngest age group.

The MEC and LEC were differentiated based on marked cytoarchitectonic differences in postnatal animals, published previously (O’Reilly et al., 2015). In short, an acellular Layer IV, named lamina dissecans, clearly separates superficial layers (II–III) from deep layers (V–VI). The lamina dissecans is particularly well developed in MEC and less clear in LEC. CB+ neurons of Layers II generate dendritic bundles that rupture the continuation of Layer II in MEC, whereas in LEC these CB neurons exhibit a more laminar organization. In contrast, CR+ neuropil was more prominent in Layer I in LEC and this labeling decreased when entering MEC (data not illustrated).

Once we established how the borders of POR change during postnatal development, Nissl staining was sufficient to determine the injection site of the tracing study in most of the cases. When the borders were not clear, particularly in younger pups, additional PV or CB immunostainings were conducted.

### Injection sites

To assess the development of POR projections in MEC, we injected anterograde tracers in different locations within POR of pups at different postnatal ages. Among the 112 animals used in this study, 23 animals did not survive surgery, or no injection sites were observed in POR. At the end, 89 injections were available for the anterograde study (Table 1).

**Table 1:** List of POR anterograde injections that were used for the study

	dPOR		vPOR		
Age	Superficial layers	Deeper layers	Superficial layers	Deeper layers	Number of rats per group
P3	x		x	x	1
P3			x	x	1
P3	x	x			2
P3	x				2
P4			x		2
P4	x	x			1
P4	x				1
P4			x	x	1
P5	x	x			1
P5		x	x	x	1
P6	x		x		1
P6	x	x			1
P6			x	x	1
P7	x		x		1
P7	x	x			1
P8			x	x	2
P9			x		3
P9			x	x	2
P10	x	x			1
P10			x		2
P11			x		2
P11	x				1
P12			x		1
P12		x			2
P12				x	1
P12	x	x			1
P13				x	1
P13		x		x	1
P13			x		1
P13	x				2
P14			x	x	1
P15			x	x	2
P15	x				1
P15		x			1
P17			x	x	1
P17	x	x			2
P17			x		1
P18			x	x	2
P18	x				1
P19			x		2
P20	x	x			2
P20			x	x	2
P20			x		2
P20					1
P21				x	3
P21			x	x	2
P21	x	x	x	x	1
P21	x	x			2
P21	x				1
P22			x		2
P22			x	x	2
P22	x	x			2
P23			x		2
P23	x	x			2
P23			x	x	3
P24		x			2
P24			x	x	1
P25	x	x			1
P31			x		1

**Figure 4.**
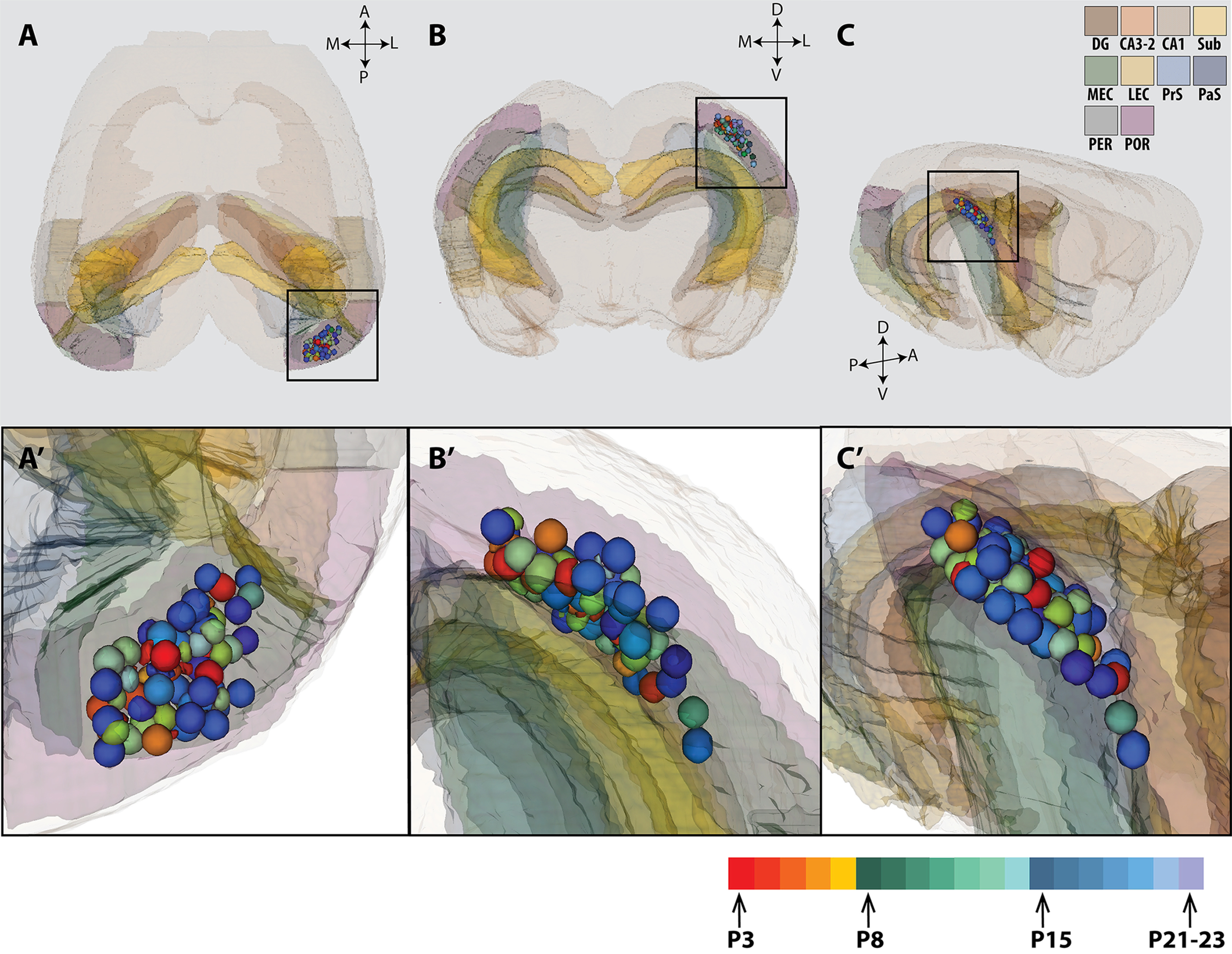
3D visualization of the anterograde injections in POR of rat pups. A total of 89 BDA injections in POR of rat pups between 3 and 23 d of age (P3–P23) were analyzed. The location of the BDA injections was normalized to a standard 3D atlas of the rat brain (Waxholm space; Papp et al., 2014). Dorsal (***A***), posterior (***B***), and posterolateral view (***C***) of the 3D atlas brain with the center of each injection (colored spheres). ***A’***, ***B’***, ***C’***, Higher magnification views of the center of the injections in POR indicated by the black square in ***A***–***C***, respectively. The color code indicates a different postnatal age (see color code bottom right). Injections were performed either in the left or right hemisphere but were all plotted in the right hemisphere here for illustrative purpose. Dentate gyrus (DG), CA3-1, subiculum (SUB), PrS and PaS, MEC and LEC, A35-36, and POR are color coded (see code in the upper right corner), and the rest of the brain is shown in transparent light brown. A: anterior; D: dorsal; M: medial; L: lateral; P: posterior; V: ventral.

**Figure 5.**
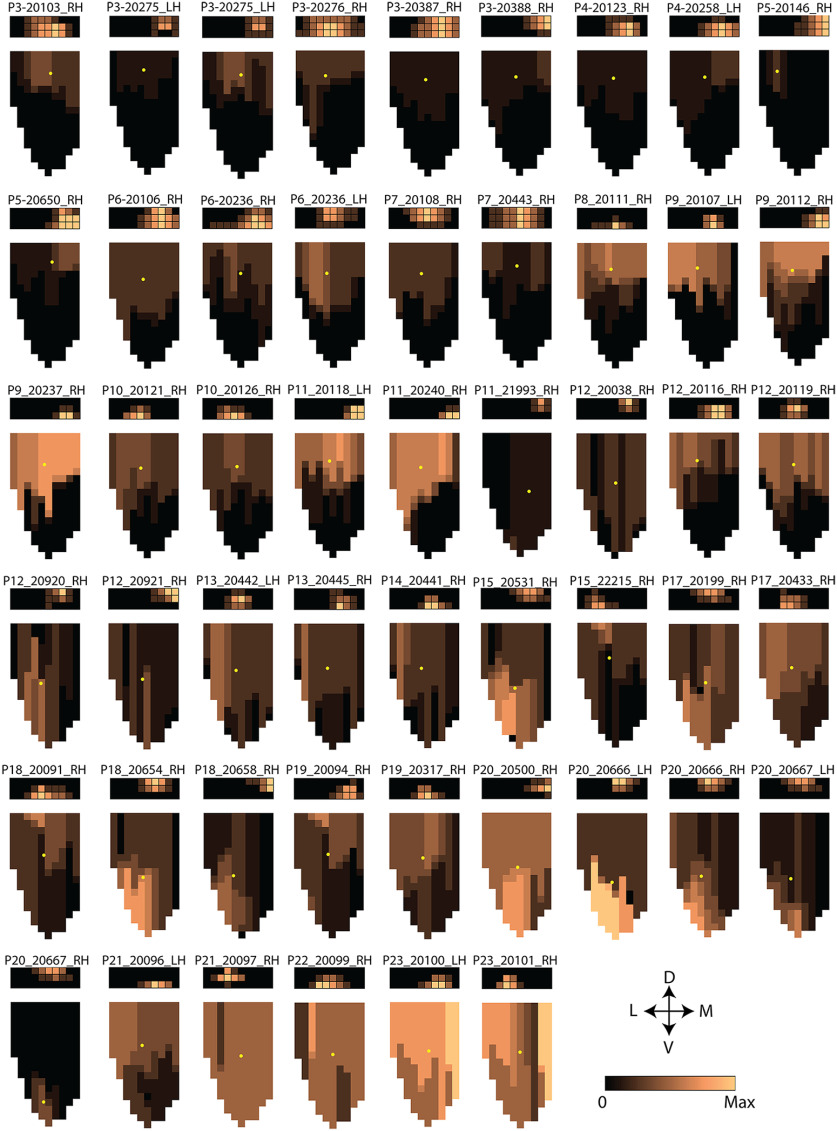
Individual flatmaps of POR with injection sites and MEC with corresponding axonal distribution and density. Individual flatmaps of POR and MEC of the 51 cases used in the quantitative analysis. The rectangle on the top represents POR, the place, and the size of the injection site along the dorsoventral and mediolateral axes as well as the relative intensity are indicated. The 2D representation below each POR represents MEC and the different colors represent the intensity of labeled fibers. The same color code (see color code on the bottom right) was used to indicate the density of labeling in POR and MEC. The yellow dot depicts the center of mass of the labeled axonal plexus in each MEC flatmap from P3 until P23. D: dorsal; V: ventral; M: medial; L: lateral.

**Figure 6.**
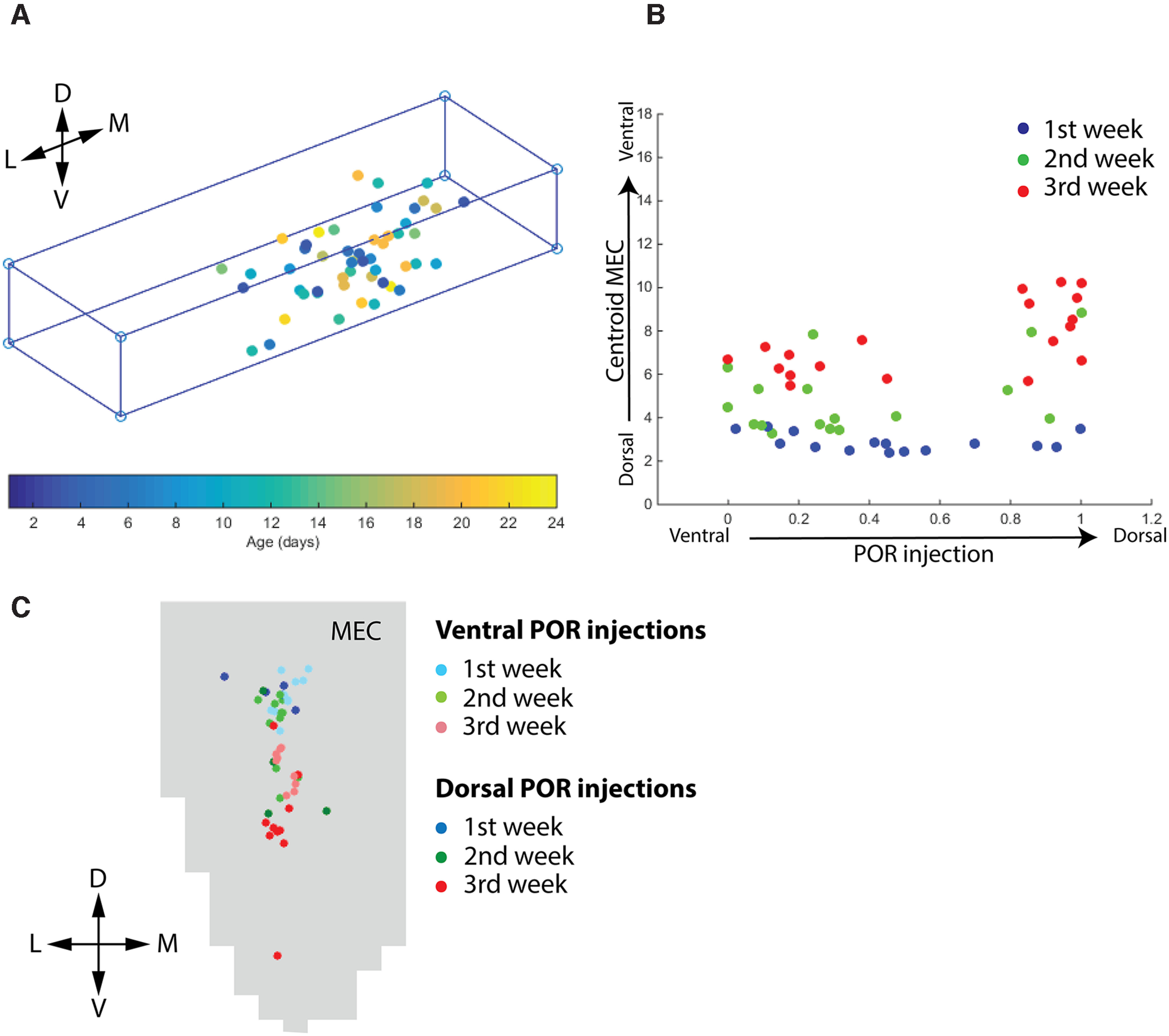
Quantitative analysis of POR injections and MEC projections centers of mass. ***A***, Normalized 3D representation of the injection sites of BDA in POR. The 3D POR structure is converted to a rectangular 3D normalized representation to obtain relative mediolateral and dorsoventral positions of the injections. The injections are color-coded by age (see bar code at the bottom of ***A***). ***B***, Normalized 2D chart of the dorsoventral position of the center of mass of the labeled axonal plexus in MEC (*y*-axis) versus the dorsoventral position of the injections (*x*-axis). They are sorted by the postnatal week (first, second, and third). Pearson’s *R* values: first week, *R* = 0.27, *p* = 0.33; second week, *R* = 0.442, *p* = 0.076; third week, *R* = 0.648, *p* = 0.003. ***C***, Normalized flatmap showing the center of mass of the labeled axonal plexus in MEC for each analyzed POR injection during the first, second, and third week of postnatal development. Injections are differentiated into dorsal POR and ventral POR injections. D: dorsal; V: ventral; L: lateral; M: medial.

To compare the location of injections in brains of different ages, we age-normalized the position of the injections in a 3D Atlas Brain (https://www.nitrc.org/projects/whs-sd-atlas/; Fig. 4). This allowed us to read out normalized coordinates in 3D, which were subsequently plotted in a flattened representation of POR and MEC (Figs. 1 and 5). The injections were mainly located in an intermediate portion of the posteromedial part of POR. This region of POR was chosen because previous studies indicated that this portion of POR strongly projects to MEC (Burwell and Amaral, 1998b; Koganezawa et al., 2015). To assess whether our dataset allowed to generalize the obtained data as representative for POR, we conducted a statistical assessment of the correlation between injection site and projection patterns. This showed that there was no significant correlation between the center of mass of the POR-to-MEC projection and the studied injection sites along the long axis of POR (data not shown). In contrast, there was a significant correlation between the position of the injection site along the dorsoventral axis of POR and the resulting labeling distribution in MEC (Pearson’s *R* values: first week, *R* = 0.27, *p* = 0.33; second week, *R* = 0.442, *p* = 0.076; third week, *R* = 0.648, *p* = 0.003). In another words, the distribution pattern of the POR-MEC projection is more sensitive to its origin along the dorsoventral axis than its lateromedial origin (Figs. 5 and 6). This was in line with the overall topographical organization described previously in the adult rat (Burwell and Amaral, 1998b; Koganezawa et al., 2015), that ventral injections of POR resulted in labeling in dorsal MEC, whereas the fibers of dorsal POR project to more ventral levels in MEC. This topographical organization was however not yet clear during the first postnatal week but became clearer during the second and third week (Fig. 6).

### Postnatal development of dorsal POR projections

**Figure 7.**
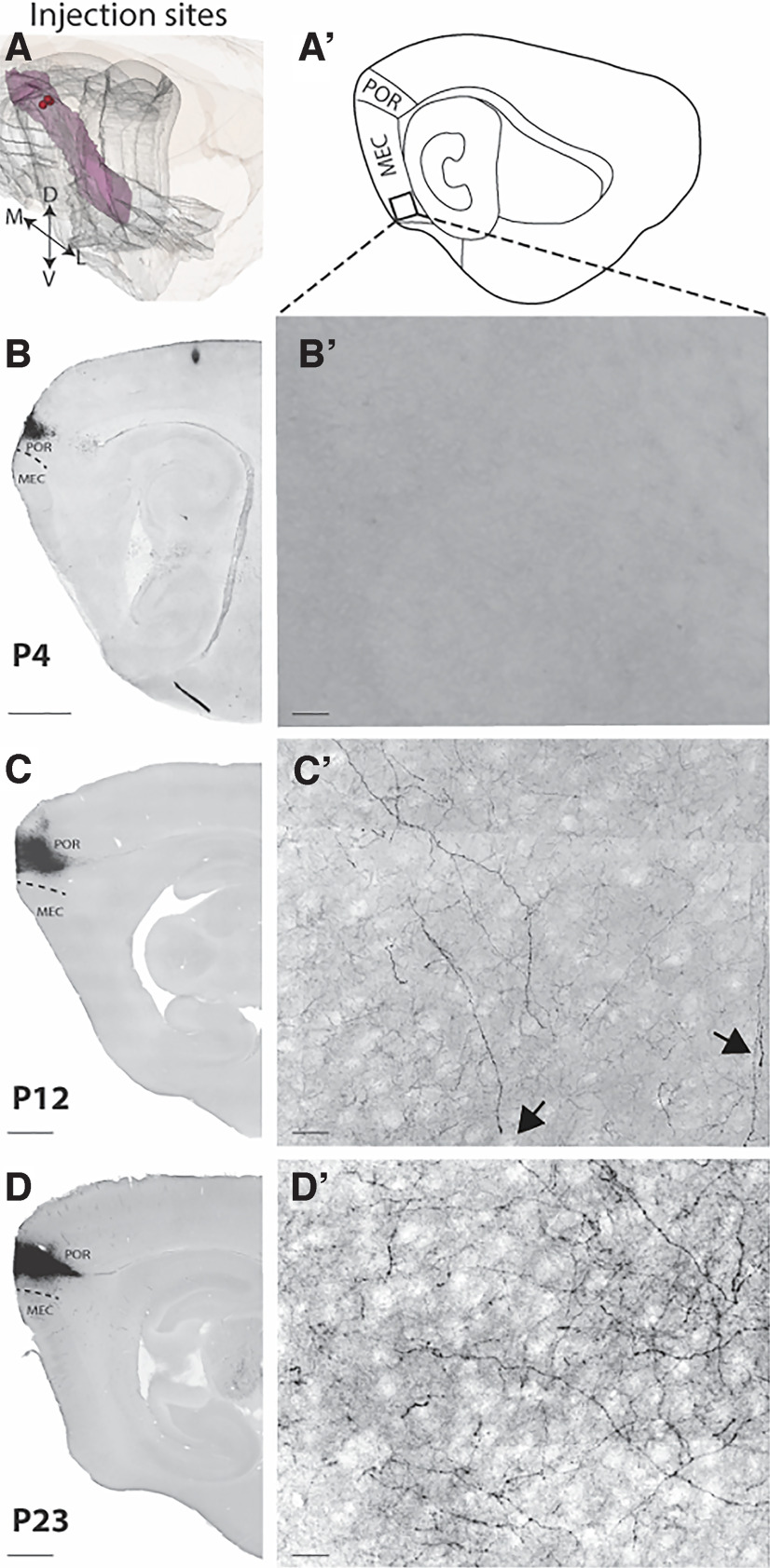
Projections from dorsal POR to ventral MEC are emerging around P12. Sagittal sections showing three representative examples of BDA anterograde tracer injections in dorsal POR at three different age points (P4, P12, and P23). ***A***, location of center of the three injections in a standard 3D atlas of the rat brain (Waxholm space; Papp et al., 2014). ***A’***, Schematic line drawing of a sagittal section illustrating the sagittal level where the three high-magnification pictures (***B’–D’***) were taken. The black square depicts the exact field where the pictures were acquired. ***B***–***D***, Pictures of the injection site in dorsal POR at the first week (P4), second week (P12), and third week (P23) of postnatal development, respectively. ***B’–D’***, Pictures in the ventral MEC showing the plexus development from P3 until P23. MEC: medial EC; POR: postrhinal cortex. Scale bar: 200 μm (***B***–***D***) and 20 μm (***B’***–***D’***).

The overall analysis as presented in Figure 6 indicated that projections from dPOR to MEC were quite immature until the end of the first week of postnatal development (Fig. 7). During the first week (P2–P7), only a few fibers were observed in the dorsal region of MEC, and no fibers were found in the ventral MEC of the rat pups (Fig. 7*B*). After the second week, fibers gradually started to appear in ventral MEC. Although the number of fibers was still far from adult-like, already at P8–P9, the topological organization of these fibers was like that seen in adult rats (Fig. 7*C*). From the beginning of the second week to the end of the third week, the density, branching, and complexity of the labeled plexus increased. During the third week of postnatal development, density of fibers increased appreciably in ventral MEC and by P24, the plexus had the typical adult like appearance (Fig. 7*D*).

Looking at the mediolateral distribution of the dPOR-MEC projection at P2–P3, we observed that dPOR axons already covered the whole mediolateral axis of MEC and this did not change with increased postnatal age. In the case of the younger rat pups, we found only a few fibers per section, and they were in the most dorsal portion of MEC, slightly more lateral than the center of mass of the injection site. This positional relationship between center of mass of the injection and the mediolateral position of the labeled axonal plexus in MEC was maintained, though the density of fibers was less in the younger than in the older rats (Figs. 5 and 7).

### Postnatal development of ventral POR projections

**Figure 8.**
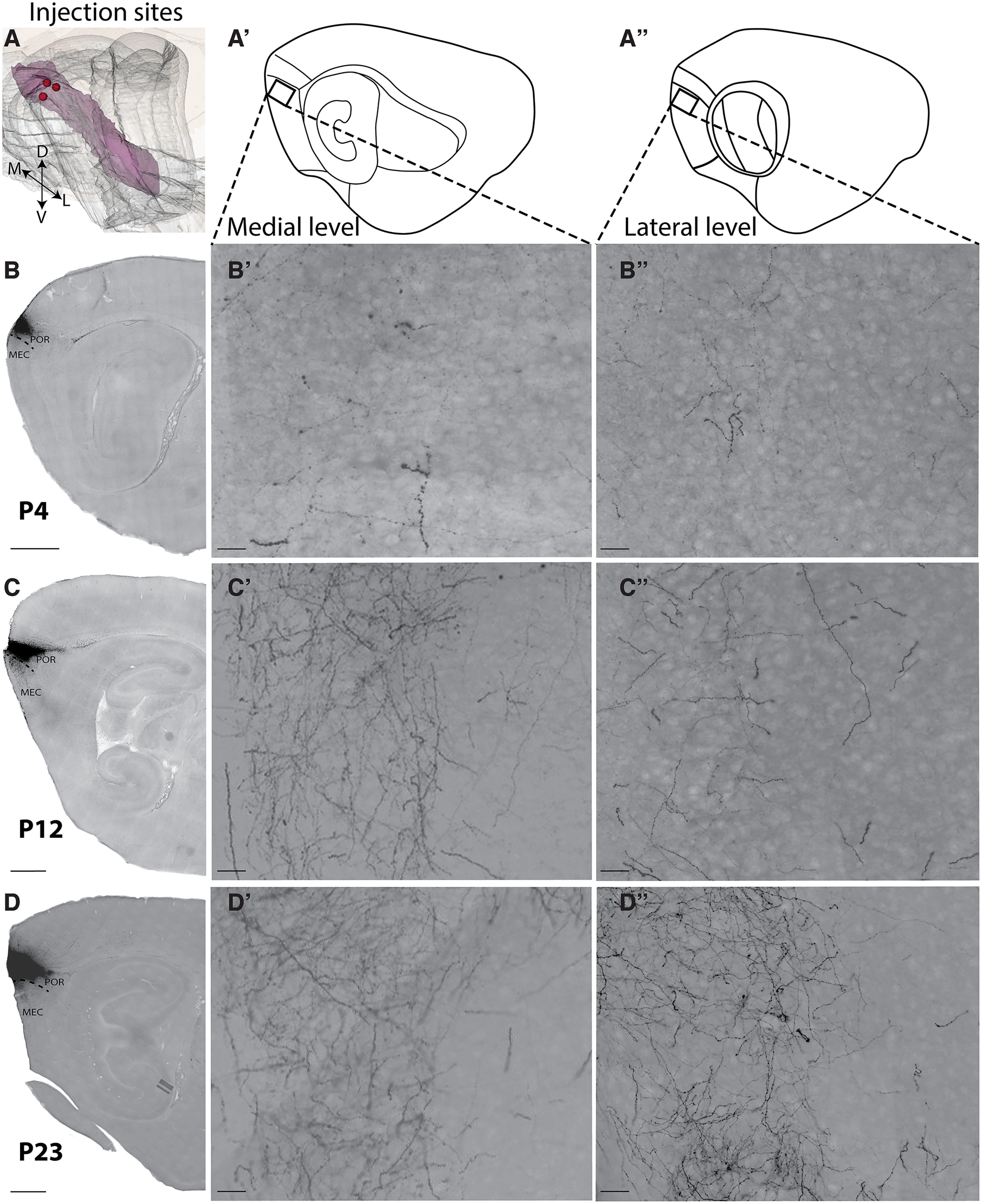
Projections from ventral POR to dorsal MEC emerge in the first postnatal week. Sagittal sections showing representative examples of BDA anterograde tracer injections in ventral POR at three different postnatal ages (P4, P12, and P23). Anterograde tracer injections in ventral POR labeled projections terminating in dorsal MEC. ***A***, Location of center of the three injections in a standard 3D atlas of the rat brain (Waxholm space; Papp et al., 2014). ***A’***, ***A’’***, Schematic illustrations of the two sagittal levels where the six high-magnification pictures (***B’***, ***B’’***, ***C’***, ***C’’***, ***D’***, ***D'’***) were taken. The black square depicts the exact field where the pictures were acquired. ***B–D***, Pictures of the injection sites in ventral POR at the first week, second week, and third week of postnatal development, respectively (P4, P12, and P23). ***B’***, ***B’’***, ***C’***, ***C’’***, ***D’***, ***D’’***, High-magnification pictures in the dorsal MEC showing the development of plexus from P4 until P23. Scale bar: 200 μm (***B***, ***C*** and ***D***) and 20 μm (***C’***, ***C’’***, ***D’***, ***D’’***).

The analysis of anterograde injections in vPOR showed that an adult-like topography of vPOR-MEC projection was already present by P2–P3. At that age, fibers arising from vPOR reached dorsal MEC and similar to what was described in case of dPOR injections, fibers distribute to parts of MEC positioned slightly lateral to the core of the injection site in POR. From P2–P3 to P23, there was a noticeable increase in the density of the labeled plexus in dorsal MEC (Figs. 5 and 8). In the first week of postnatal development, scattered fibers were found along the mediolateral and the dorsolateral axis of MEC. During the second week, the density of fibers increased, and the plexus became more complex because of an increased collateralization of axons. From P3 until P14, the density of labeling gradually increased from the dorsal portion to the more ventral portion of MEC and from more medial to more lateral portions of MEC (Fig. 8). In the first postnatal week and beginning of the second postnatal week, there were more labeled fibers close to the injection, i.e., in the sagittal sections containing the injection site, than in more lateral or medial sections. Nevertheless, from the end of the second week until P23, labeling increased in density and spread along both the mediolateral and dorsoventral axes of MEC.

As mentioned above, the PaS may be positioned in between MEC and dPOR. This can be easily missed, particularly in young postnatal animals since the areas are not easily differentiated from each other. It is therefore that we have not always been able to exclude minor involvement of PaS in our anterograde tracer injections, explaining the occasional strong labeling in Layer II of dorsal MEC, which is likely the result of PaS involvement (Doan et al., 2019). We cannot exclude that relatively dense projections in dorsal MEC might exist in the first postnatal days since axons are still growing toward their more ventral targets or alternatively, they could be pruned during postnatal development. The latter explanation is however not very likely in view of the commonly reported observation that connectional development within the parahippocampal-hippocampal system does not seem to be governed by pruning (O’Reilly et al., 2013; Haugland et al., 2019).

### Postnatal development of center of mass of the POR-MEC projections

To quantitatively assess the distribution of labeling in MEC in relation to developmental age and origin in dPOR or vPOR, we selected a representative set of 51 out of the 89 injections that we used for the qualitative analysis of the POR-MEC projection described above, and separately analyzed dPOR (*n* = 25) and vPOR (*n* = 26) injections (see Materials and Methods for selection criteria).

**Figure 9.**
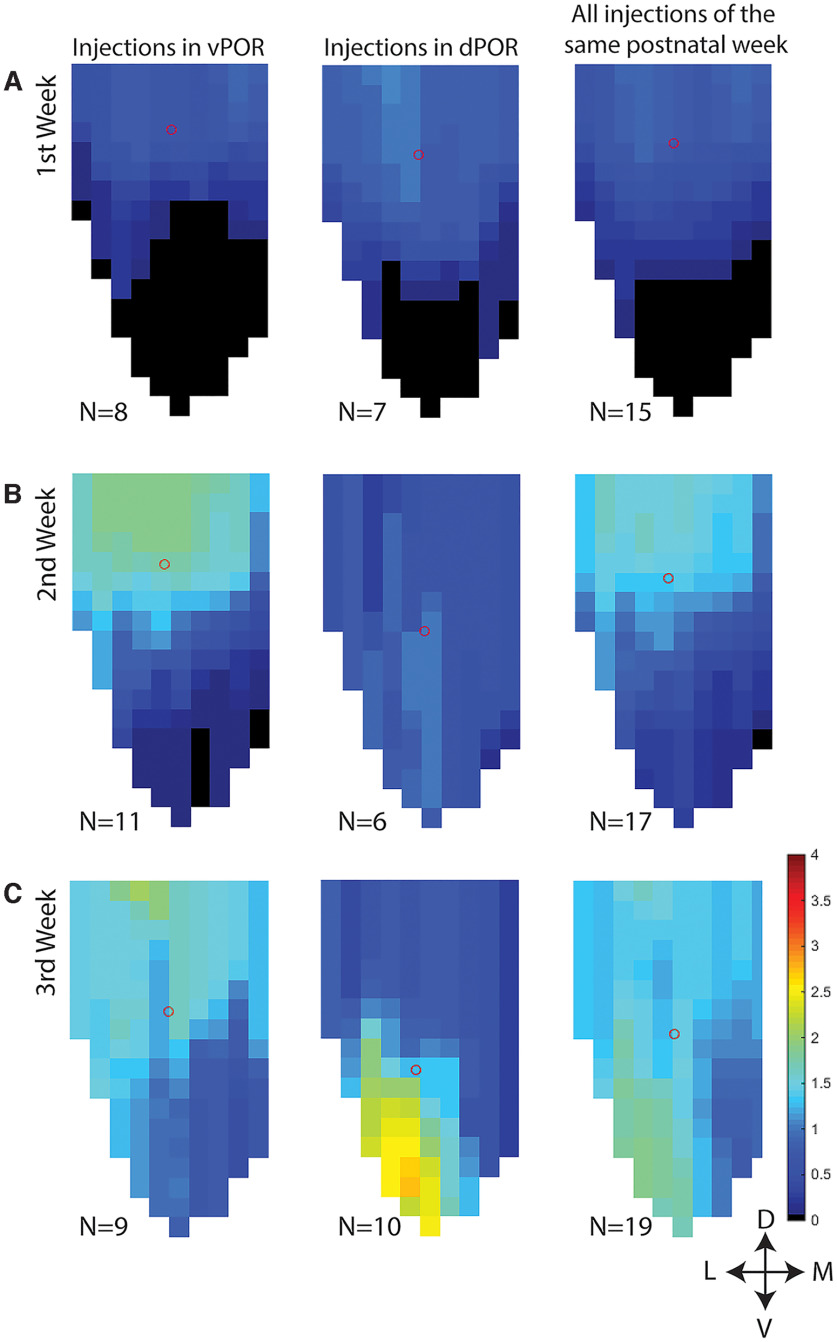
Flatmaps of POR projections in MEC across age groups. The injections were divided into three groups based on the age of the injected animal. ***A***, Flatmaps of averaged labeling patterns in animals injected during the first postnatal week. ***B***, Flatmaps of animals injected during the second postnatal week. ***C***, Flatmaps of animals injected during the third postnatal week. The first column shows flatmaps with the distribution of the vPOR-MEC projection, the second column shows flatmaps with the distribution of the dPOR-MEC projection. The third column shows flatmaps with distribution of the fibers independent of the location of the injection site in POR. The number of used animals is indicated directly below each flatmap(*N*). The density of the fibers is depicted by a color code; see color bar in the right bottom of the figure. The circle shows the center of mass of the POR-MEC projections. D: dorsal; V: ventral; L: lateral; M: medial.

We produced normalized flatmaps of the location of the labeling in each experiment (see Materials and Methods and Figure 1 for details). These flatmaps consisted of a matrix of bins spanning the dorsoventral and mediolateral axis of MEC (Fig. 9). Each bin was assigned a value between 0 (black) and 4 (red), reflecting the relative density of labeled fibers. A first comparison of the obtained flatmaps across animals of different ages corroborated the observations described above. Injections in vPOR in young animals resulted in labeling with an overall more dorsal position in MEC than in the older ones. Likewise, dPOR-MEC projections in young animals were confined more dorsally in MEC, whereas they extend into the most ventral portion of MEC in the older animals (Fig. 9).

To quantify this, we calculated the center of mass of the labeled plexus, and we tested the relationships between this measure and the coordinates of the injection sites and the age of the animal (*n* = 51). We performed a k-means cluster (two clusters: dPOR and vPOR; maximum iterations = 10) and the results indicated that the data can be separated into two clusters of 23 and 28 cases respectively (one-way ANOVA; *p* < 0.001). Next, we divided our set of injections into two injection site groups, dPOR and vPOR injections based on anatomic criteria. This resulted in 23 vPOR-injections and 28 dPOR-injections, corroborating the results of k-mean cluster analysis. We next tested whether the injection site in POR has an effect on the position of the plexus in MEC with one-way ANOVA with the injection site (dPOR and vPOR) as independent variable, and the center of mass of POR-MEC projection as dependent variable. This showed a significant effect (*p* < 0.001). Later we investigated whether this differential POR-MEC projection pattern was present from the first week or developed later during the postnatal development (second and third postnatal week). One-way ANOVA analysis showed that the location of the centers of mass of the labeled plexus was not significantly different between the two clusters (dPOR injections and vPOR injections) in the first week of postnatal development (*n* = 15; *p* = 0.167). However, in the second week of postnatal development, the centers of mass of the labeling in MEC were significantly more dorsally located in case of vPOR injections than dPOR injections (*n* = 17; *p* = 0.005). This statistical difference became even larger in the third week of postnatal development (*n* = 19; *p* = 0.001). This thus indicates that dPOR projections reached more ventral portions of MEC in older animals compared with dPOR injections in younger animals and compared with vPOR injections in animals of the same age. The topographical change of the POR-MEC projection during development was further confirmed by a two-way ANOVA on the effect of age and injection site on the position of the plexus in MEC. This showed a significant effect of age (*p* <0.0001), injection site (*p* < 0.0001), whereas the interaction age and injection site was barely significant (*p* = 0.052).

Additionally, we assessed whether the labeling patterns in MEC changed systematically in relation to the mediolateral and the dorsoventral position of the injections in POR. Statistical analysis showed no clear relationship between mediolateral placement of the injection site and the center of mass of the labeled axon plexus (*n* = 54; mediolateral position of the injection site vs center of mass in MEC along the mediolateral axis: *R* = 0.12; *p* = 0.42; mediolateral axis of the injection site vs center of mass in MEC in the dorsoventral axis: *R* = 0.19; *p* = 0.18). In contrast, there was a significant relationship between the dorsoventral placement of the injection and the dorsoventral location of the center of mass of labeled axon terminals in MEC (*n* = 51; *R* = 0.40; *p* < 0.0001). More dorsal POR injections resulted in centers of mass of the labeled plexus that were located more ventrally in MEC, and more ventrally placed injections resulted in labeled plexus with a center of mass located more dorsally in MEC. However, the dorsoventral placement of injections was not significantly related to the mediolateral location of the centers of mass of the projections (*R* = −0.084; *p* = 0.558).

### POR neurons projecting to MEC

**Figure 10.**
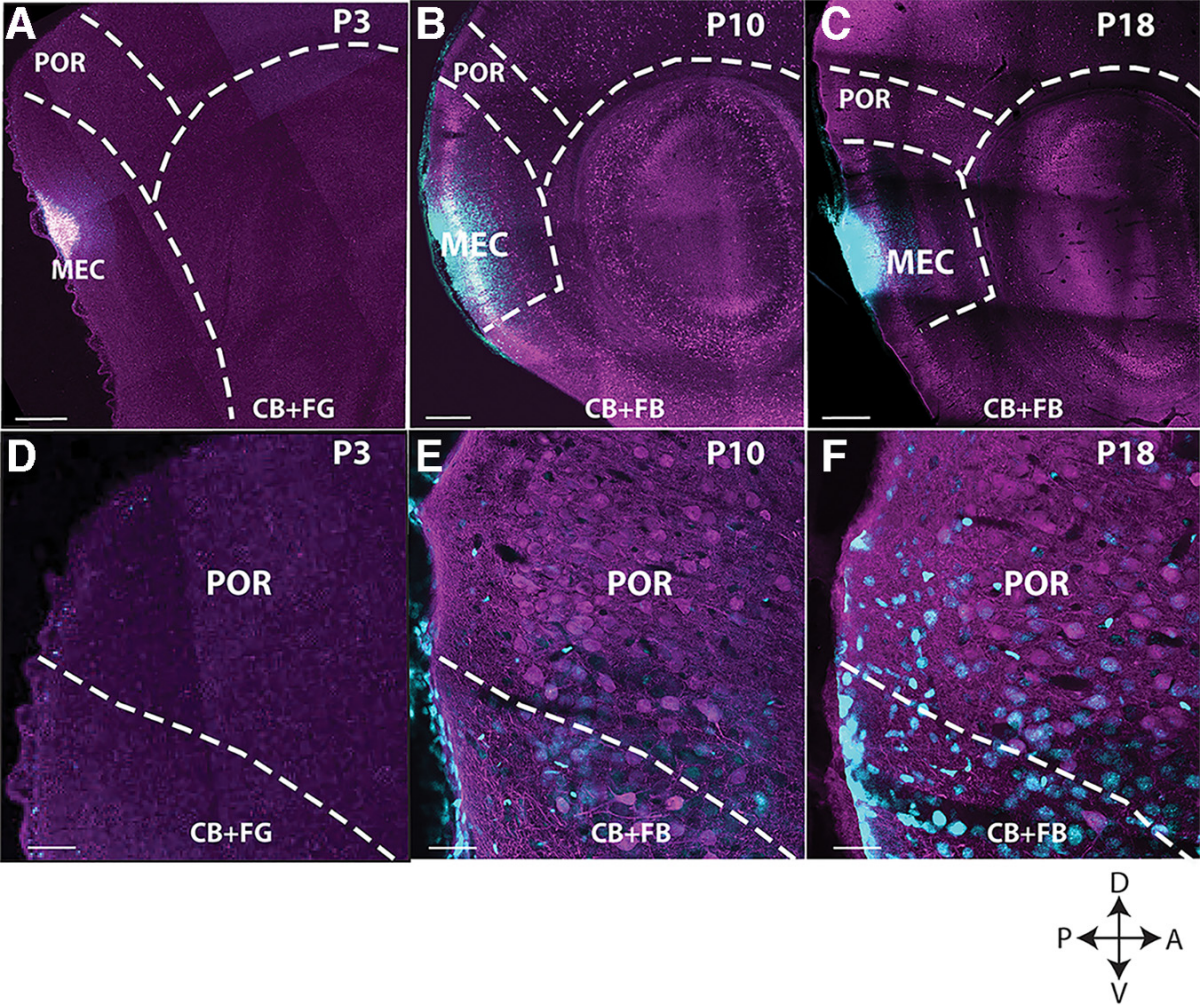
Retrograde tracing from intermediate dorsoventral levels of MEC at three postnatal stages. Retrograde injections in rat pup brains P3–P5, P10–P12, and P18–P23. ***A–C***, Low-power sagittal images showing an example of the injection site in MEC at P3, P10, and P18, respectively. ***D***–***F***, High-power images, showing retrogradely labeled projecting neurons (cyan) and CB-positive neurons (magenta) in POR. At P3–P5, there are barely positive MEC-projecting neurons in POR (***D***). in older animals (P10; ***E*** and P18; ***F***), gradually more neurons in POR are labeled. Note that in the P3 animal, only very weak CB labeling is present (see also Fig. 2). CB: calbindin, FB: fast blue. Scale bar: 500 μm (***A***–***C***) and 20 μm (***D***–***F***). D: dorsal; V: ventral; A: anterior; P: posterior.

The anterograde tracing data led to two conclusions. First, the POR-MEC projections developed gradually from the first postnatal week until the end of the third postnatal week, showing increasing density and branching complexity. Second, the POR-MEC projections did not reach the ventral MEC until the end of the first postnatal week. With the use of retrograde tracing experiments, we corroborated these observations, by showing a relation between postnatal age and the overall density of labeled projecting neurons in POR. We compared the labeled neurons in POR following injections in intermediate-ventral MEC at P3–P5, P10–P12, and P18–P23 (*n* = 13). Retrograde tracer injections at P3–P5 in the intermediate portion of MEC did not result in labeled neurons in POR (Fig. 10*A*,*D*). In MEC, retrogradely labeled neurons were noticeable, indicating that tracer uptake and transport did take place. In contrast, injections in intermediate MEC at P10–P12 did result in some retrogradely labeled neurons in POR (Fig. 10*B*,*E*). In the third week, increased retrograde labeling was observed and from P18 onward the number of labeled neurons stabilized (Fig. 10*C*,*F*).

### Maturation of MEC

To evaluate the synaptic maturation in MEC, immunohistochemistry for two synaptic proteins, synaptophysin and bassoon, was conducted in sections obtained from brains during the first, second and third week of postnatal development (*N* = 10).

**Figure 11.**
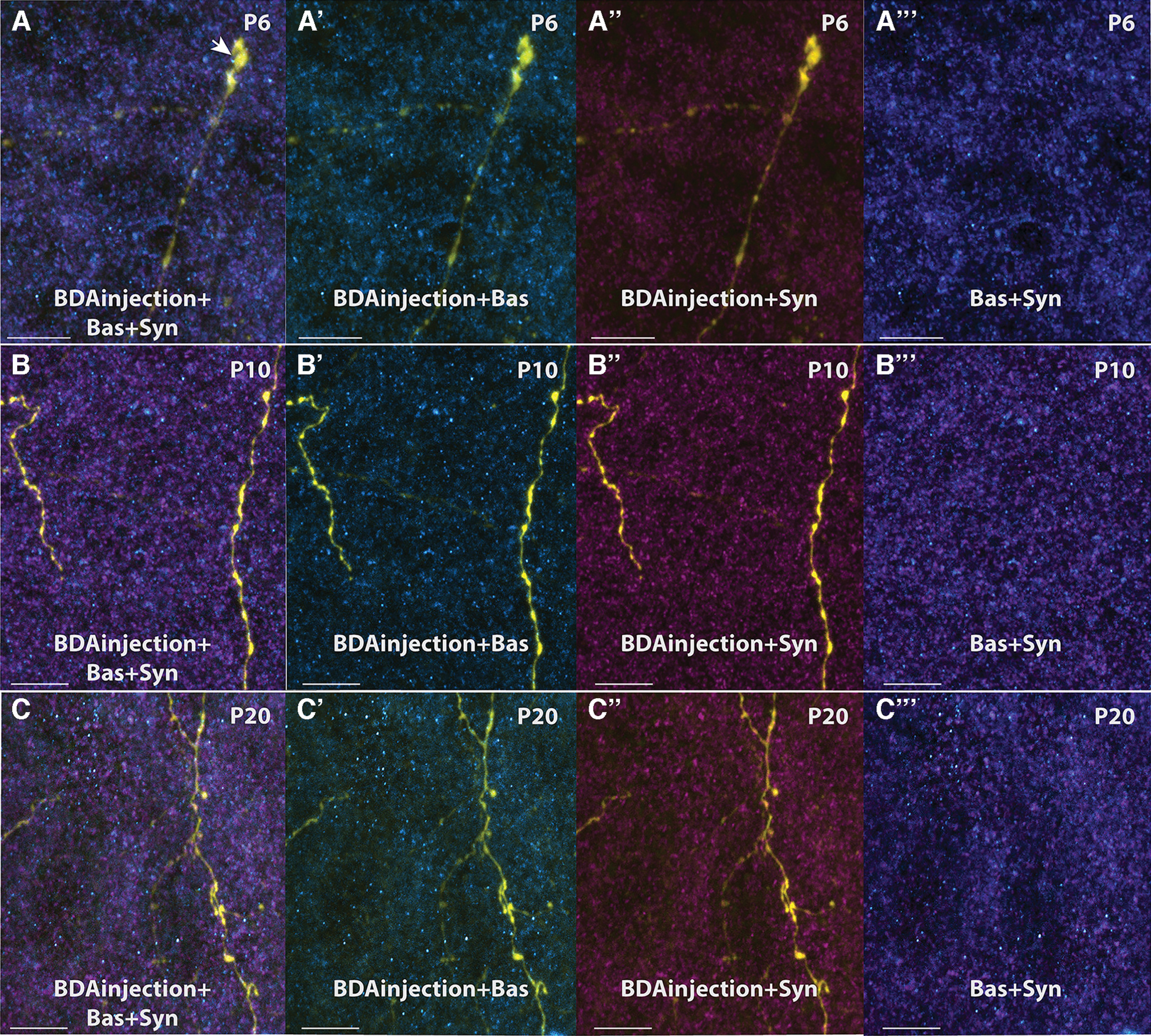
Immunolabeling for bassoon and synaptophysin shows postnatal synaptic maturation of BDA-labeled POR-axons in MEC. Confocal images were taken in dorsal MEC at P6 (***A***, ***A’***, ***A’’***, ***A’’’***), P10 (***B***, ***B’***, ***B’’***, ***B’’’***), and P20 (***C***, ***C’***, ***C’’***, ***C’’’***). They show a change of the bassoon and synaptophysin labeling as well as changes in the maturation of the fibers coming from POR. In the younger rats, bassoon and synaptophysin labeling is more diffuse than in the older rats. Growth cones were observed more often in the first week of development than in second and third postnatal weeks (arrow shows an example of a growth cone). BDA labeling in yellow; Bas: bassoon in magenta; Syn: synaptophysin in cyan. Scale bar: 20 μm.

During the first week of postnatal development, bassoon immunoreactivity was widely distributed along the axons and growth cones, without showing a clear punctate pattern, considered to be typical for mature synaptic contacts (Fig. 11*A*,*A’*,*A’’’*; Dhingra et al., 1997). From the second week of postnatal development onwards, the immune staining showed a more punctate morphology, and bassoon immunoreactivity was mainly found in the protuberances of the fibers (Fig. 11*B*,*B’*,*B’’’*). Likewise, the pattern of synaptophysin labeling changed between the first week and the second week of postnatal development. In the first postnatal week, synaptophysin staining was distributed along the fibers and the labeling did not show a clear punctate structure (Fig. 11*A*,*A'’*,*A’’’*). It was only in the second postnatal week that the labeling of synaptophysin started to allocate into the protuberances of the fibers showing a clear puncta-like structure. Changes between the labeling patters in the second versus the third postnatal week are less dramatic (Fig. 11), indicating that synaptic maturation is prominent in the second postnatal week. This conclusion is in line with the observations that the number of growth cones decreases over time. At P3, we identified more than four growth cones in every section. At the end of the second week, growth cones were still observed, but not in all sections and if present, the number of growth cones per section was less than four. By the beginning of the third week, POR-MEC projections already were quite adult-like; growth cones were rarely seen and the synaptic staining with bassoon and synaptophysin is exclusively punctate mainly present in the endpoints of collaterals.

**Figure 12.**
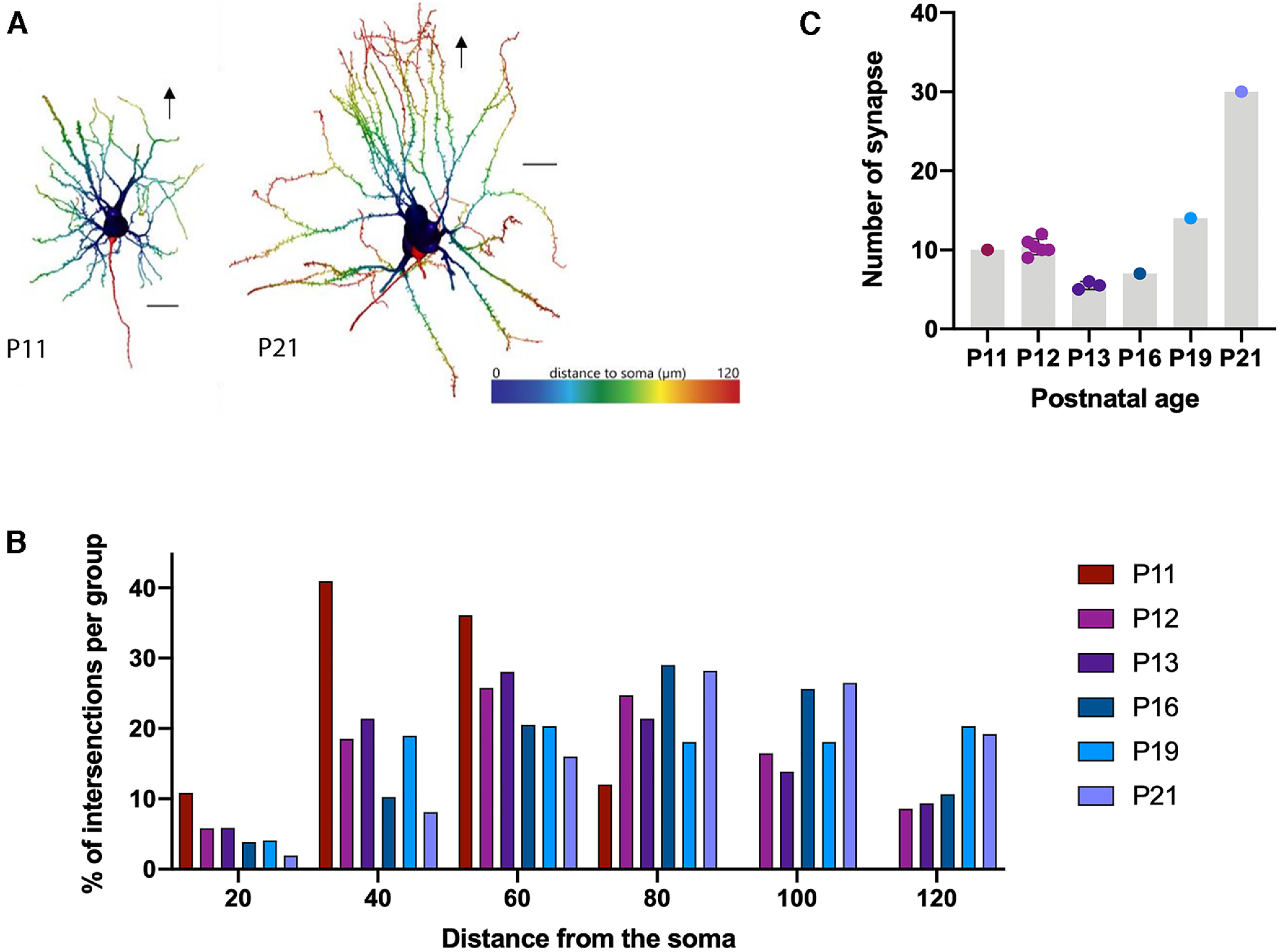
Increasing complexity of superficial MEC dendritic tree. ***A***, Reconstructed MEC Layer II principal neurons showing their complexity in a 3D representation. On the left a P11 neuron and on the right a P21 neuron. The arrow indicates the direction to the pial surface, Layer I. The distance from the soma along the dendrites is indicated by the heat map. The dark blue represents the soma (distance, 0 μm), gradually shifting to red, which represents the most distal part of the dendritic tree related to the soma. Scale bar: 20 μm. ***B***, Sholl analyses demonstrate changes in the dendritic branching pattern of the reconstructed MEC LII (number of intersections) as a function of the radial distance from the soma (μm). ***C***, Quantification of the number of synapses between LII neurons and POR projections in MEC. Each dot represents one measure.

Analysis of the intracellularly filled Layer II neurons in MEC supported the former results. The 3D reconstruction of the neurons (see Fig. 2) showed that the complexity of the dendritic trees increased as measured by the increasing overall length and the distribution of numbers of intersections as a function of distance from the soma and age (Fig. 12*A*,*B*). The number of synapses also increased between the second and the third postnatal week (Fig. 12*C*).

### Development of functional connectivity from POR to MEC

**Figure 13.**
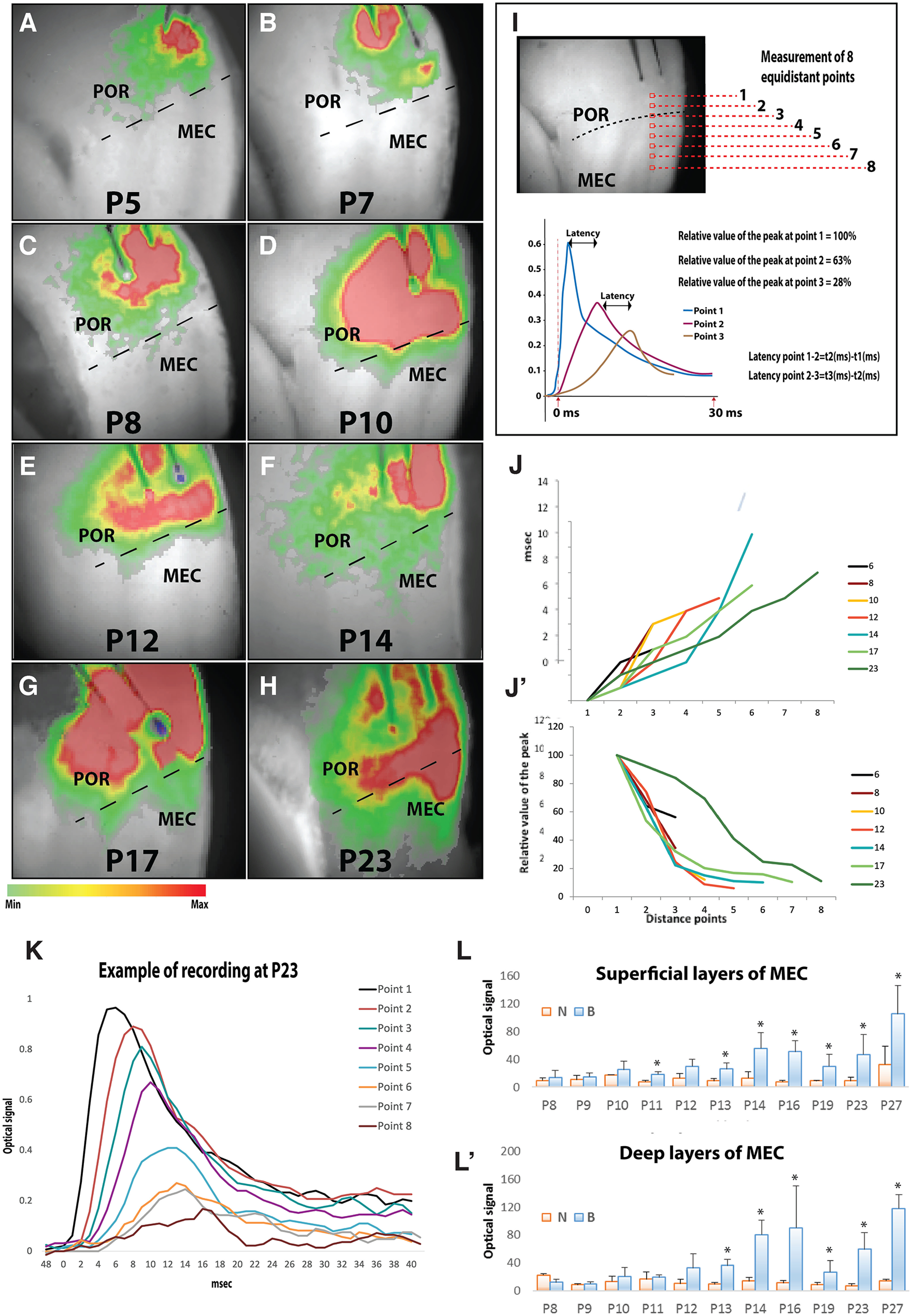
VSD-imaging recordings during postnatal development shows increased spread and strengths of POR projections during postnatal development. ***A–H***, Representative samples of VSD-imaging, starting at 13 ms after stimulation, at different postnatal ages. The colored bar below depicts the relative strength of the signal. ***I***, To quantitatively assess the spread of the signal, eight equidistant points were measured in the different slices. Two of them are located in POR, one around the border between POR and MEC, and five are in MEC. The red dashed line and the small red square depict where the eight measurements were recorded (numbered 1–8). The graph with the three peaks shows examples of how the latency was measured. ***J***, ***J’***, Values of the latency (refers to how much time, measured in milliseconds, a signal takes to travel from one point to the following point) and relative value of the peak amplitude versus distance respectively. Points 1–8 in panels ***J*** and ***J’*** correlate with points 1–8 in ***I***. ***K***, Example of the recorded optical signal at each of the eight points that were used for the analysis in a 23 days old pup rat (P23). The *x*-axis depicts msec after stimulation. ***L***, ***L’***, Effect of the GABAA receptor antagonist, bicuculline. POR fibers were stimulated and the optical signal in superficial and deep layers of MEC was measured before (N) and after (B) adding bicuculline; *significant difference, *p* ≤ 0.05.

As we have described above, POR fibers already reached the dorsal portion of MEC from P3 onwards. However, the density of these fibers, and how far they extended to ventral levels of MEC increased from the first week until the third week of postnatal development. A similar developmental gradient was observed in development of the dendritic extent and complexity as well as the staining for synaptic markers, indicative for established functional connections. As a next step, we evaluated the development of functional connectivity between POR and MEC with the use of VSD imaging. In sagittal slices through the POR-MEC region of the brain, we electrically stimulated POR and analyzed whether and with what spatial pattern the electrical signal propagated from the site of stimulation into MEC (Fig. 13).

In 261 slices, taken from 48 animals between P3 and P27, we stimulated Layer II/III or/and V/VI of POR. In each animal, we recorded from all sagittal slices that contained POR and MEC. During the first postnatal week, the signal did not reach MEC, in any of the analyzed slices, and the optical signal was always confined to the area close to the stimulation site in POR (Fig. 13*A*,*B*). However, by P8–P9, the stimulation of POR evoked activity in MEC in a few cases in which the electrical signal slightly passed the POR/MEC border (Fig. 13*C*). From P9 to P10 onwards, the chances of excitatory responses in MEC after POR electrical stimulation increased (Fig. 13*D–H*). Stimulation of POR evoked electrical responses in MEC at P9–P12 in an area close to the border with POR. In older animals, the spread of the signal gradually extended more ventrally from the POR/MEC border into MEC (Fig. 13*E–H*).

To quantitatively assess the propagation of the electrical signal from POR to MEC, we calculated the average value of the maximum amplitude of the peak at different ages from P6 to P23. In each slice, the maximum amplitude was measured at eight equidistant points from the onset of the signal in POR into MEC (Fig. 13*I*). The two first measurements were in POR (Fig. 13*I*, points 1 and 2), one was around the POR/MEC border (point 3), and five were in MEC (point four until point 8). This analysis confirmed the qualitative observation that before P8, electrical stimulation of POR did not evoke response in MEC (Fig. 13*J*,*J’*). The differences in latencies between each of the subsequent measuring points (as explained in Fig. 13*I*,*K*) indicated that latencies and thus speed of propagation of the excitatory signal does not change during this postnatal development [Fig. 13*J*; P6 (*n* = 3) = 0.57 ± 0.23 ms; P8 (*n* = 3) = 0.38 ± 0.18 ms; P10 (*n* = 4) = 0.42 ± 0.28 ms; P12 (*n* = 3) = 0.53 ± 0.31 ms; P14 (*n* = 3) = 0.69 ± 0.35 ms; P17 (*n* = 4) = 0.83 ± 0.33; P23 (*n* = 3) = 0.98 ± 0.34 ms]. Moreover, the relative decrease (100% in the value in point 1 of our measurement; Fig. 13*I*,*J’*) of the maximum amplitude value versus distance of the eight measured points show a gradual decrease in amplitude, showing a difference in the spread of the signal. There was a direct relationship between the age of the rat and the distance that the signal traveled, such that the signal propagated further ventrally in MEC in older than in the younger animals (Fig. 13*J’*), which is in line with the anatomic connectivity date presented above.

### Development of inhibitory GABA system

During cortical development in the brain of newborn rodents, around P8–P12, the postsynaptic effect from GABA shifts from depolarization to hyperpolarization. This change, accompanied by gradual changes of Cl- homeostasis has been related to the maturation of synapses (Peerboom and Wierenga, 2021). To assess whether the maturation of the GABA inhibitory system of this region is parallel to the development of the POR-MEC projection, a set of experiments with the GABAa antagonist bicuculline (5 μm) was conducted. We measured the area under the curve during the 250 ms after stimulation in POR. Measurements were taken in superficial and deeper layers of MEC before and after the application of bicuculline. We recorded in MEC from slices taken from a range of ages, P8 until P23. The starting age P8 was chosen since stimulation in POR did not evoke signals in MEC before P8, as described above. Recordings were made in the same location before and after adding bicuculline.

After applying bicuculline, the area under the curve increased significantly in the superficial layers of MEC from P11 onwards (Fig. 13*L*). In contrast, in the deeper layers of MEC this inhibitory effect of GABA system is not significant until P13 (Fig. 13*L’*). Data thus indicated that the superficial layers of MEC are sensitive to inhibitory effects of GABA before deeper layers of MEC. This onset of the so-called GABA-shift in the POR-MEC projection, starting around P11 in superficial layers of MEC, is in line with previous studies in the hippocampus where the GABA-shift occurs around the same postnatal age (Cherubini et al., 1991). In contrast, the shift in deep layers of MEC only becomes apparent at P13, suggesting a later maturation of these deep layers. This is in line with a report on the PrS/PaS-MEC projection, where the GABA-shift in superficial layers was also reported to occur earlier than in deep layers of MEC (Canto et al., 2019). The postnatal shift of the effects of GABAergic transmission in the PHR thus coincides with the maturation of the different types of spatially modulated cells in MEC, which is in line with currently accepted continuous attractor models for the generation of head direction and grid cells since these depend on a balance between excitation and inhibition (Lovett-Barron and Losonczy, 2013).

### Development of POR-LEC projections

**Figure 14.**
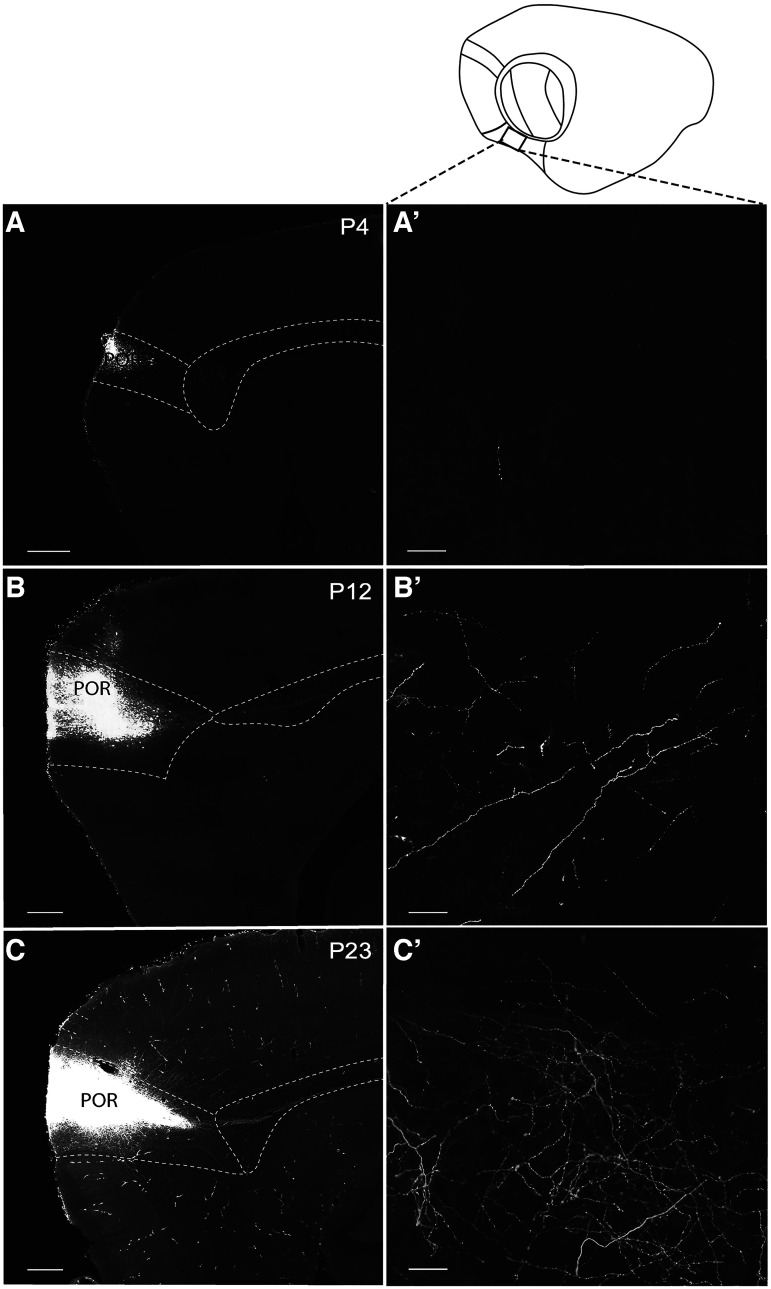
Postnatal development of POR projections in LEC. Sagittal sections showing three representative examples of BDA anterograde tracer injections in dorsal POR at three different age points (P4, P12, and P23). The POR injections are the same as in Figure 5. The square in the schematic drawing of a sagittal section at the top right depicts the exact field in LEC where the images were acquired. ***A***–***C***, Images of the injection site in dorsal POR at the first week (P4), second week (P12), and third week (P23) of postnatal development, respectively. ***A’–C’***, Images of LEC showing the plexus densities at P3, P12, and P23. Scale bar: 100 μm (***A***–***C***) and 20 μm (***A’–C’***).

In view of recent data showing that in adult rats POR also provides a substantial projection to LEC (Doan et al., 2019), we also qualitatively characterized the postnatal development of POR fibers to LEC by analyzing the same anterograde tracer injections in POR of rat pups aged P3 to P23. A systematic comparison with the patterns described above showed that POR fibers reach LEC slightly later than MEC. In all cases of the youngest pups in which we noticed the emergence of a terminal-like plexus in the most dorsal part of MEC, no fibers were observed in LEC (Fig. 14). It was only around P4–P5 that we started to see sparsely labeled fibers in LEC and a real terminal branching pattern in LEC was first seen in P8–P9 animals. From the beginning of the second postnatal week the topographical organization became like that of an adult rat, but the complexity of the axons was still rudimentary by the end of the second week. During the third postnatal week the axonal branches became more complex and by the end of the third week they reached an adult-like phenotype.

## Discussion

In this study, we describe the development of projections of POR to EC, with a focus on the projections to MEC. In adult animals, this projection follows a dorsoventral organization such that dPOR projects denser to the ventral MEC than vPOR, which shows a preference for a more dorsal innervation (Koganezawa et al., 2015; Doan et al., 2019). However, during the first postnatal week, this clear difference between vPOR and dPOR projections is not yet apparent, since both vPOR-MEC and dPOR-MEC projections reach only the most dorsal portion of MEC at P3–P5. Our tracing data, supported by *in vitro* electrophysiological data, further show that this development starts in the second half of the first week but really expands in the second half of the second week to continue at least up to P23 when it reaches an adult distribution pattern, density, and associated functionality.

### Development of the projection to MEC from P2 until P24

Although the typical adult topological organization of this projection is not clear until the start of the third week of postnatal development (from P14 onwards), POR fibers reach the most ventral portions of MEC by P6–P8 already, and the density of the fibers keeps increasing from the second week until P24. This pattern of relatively slow development of anatomic connectivity along the dorsal to ventral axis of MEC differs strikingly from what we reported previously in case of projections from PrS/PaS and from RSC. For all three inputs, fibers distribute across the entire dorsoventral extent of MEC, sparsely reaching ventral levels of MEC already from P3 (Sugar and Witter, 2016; Canto et al., 2019). However, the dPOR projections do not reach ventral levels of MEC before P6–P8. Since the methodology was comparable in these three sets of experiments, we conclude that the POR-to-MEC projection develops a bit later than PrS/PaS-to-MEC and RSC-to-MEC projections.

Our electrophysiological data show that before P7 the functional connectivity of POR is largely intrinsically confined and that it is only from P9 to P10 onwards, that POR inputs evoke responses in the MEC region close to the border with POR. We consider these POR-MEC connections to be monosynaptic as indicated by the response latency of around 8 ms in our VSD imaging experiments (Koganezawa et al., 2015). After P11 the electrical signal spreads further in time and space, suggesting an increase of monosynaptic and polysynaptic connections. This may reflect the combined effects of the progressive maturation of (1) the projections, (2) the apical dendrites of the recipient neurons, and (3) the maturation of synapses. Our study of bassoon and synaptophysin as markers for active synapses show that the number of mature synapses gradually increases from the first week until the fourth week of postnatal development across a dorsal to ventral gradient in MEC, which is in line with a previous data acquired in mice (Donato et al., 2015). This is accompanied by a parallel maturation of the MEC neuronal dendritic trees (Canto et al., 2019). Besides, we also reported an increase in synapse density of POR axons in superficial layers of MEC during the second and third week. All together, these observations may explain the increase in the electrical activity from dorsal to ventral MEC when POR is electrically stimulated in brain slices of rat pups from age P9 to P23.

### Development of the projection to LEC from P2 until P24

For many years, POR has been considered as a main, almost exclusive input to MEC (Burwell and Amaral, 1998a,b; Burwell, 2000). However, recent evidence in adult rats, corroborated by data in monkeys, indicate that POR also issues strong projections to LEC (Doan et al., 2019). This change of concept occurred after this study was designed and many of the data were collected. The added analysis of the postnatal development of the projections of POR to LEC is therefore restricted to an anatomic re-analysis of the experimental dataset. The data point to a developmental pattern, in line with what we observed for the projections in MEC, but just slightly delayed in time, which is in line with our observations that projections grow into the most dorsal levels of EC first, and over time, these projections reach more ventral parts of EC. This means that dorsal parts of MEC are innervated first, followed by more lateral and ventral parts of MEC as well as adjacent posterodorsal parts of LEC around P4. More ventral and anterior parts of LEC are innervated in a later stage around P8–P9 followed by axonal branching around P10, eventually leading to a more adult-like innervation around our last data point at P23. Although we lack electrophysiological data to assess the establishment of functional connectivity, our observations in MEC indicate that functional connectivity is established around the time that clear axonal branching is seen, accompanied by a clear morphologically visible presence of en passant and terminal varicosities. Taken together, this suggests that (1) POR projections to LEC are probably not functional before P8; and (2) the more ventral regions of LEC will be the last to receive fully mature POR projections. However, further studies are needed to corroborate this.

### Development of POR to EC projections match functional changes

Our data on the development of POR projections to EC lead to two main conclusions. First, these projections develop gradually such that functional connections with dorsal MEC emerge after the first week and they continue to develop reaching more ventral parts of MEC during the subsequent two to three postnatal weeks. Second, the projections to LEC lag a few days such that functional connections from POR to LEC might be inferred to be present by the end of the second postnatal week.

It is of interest to compare the developmental pattern in MEC reported here with the patterns reported for three other inputs to MEC, those from PrS, PaS, and RSC. These inputs show an adult-like distribution already in the beginning of the first postnatal week, i.e., when inputs from POR are just beginning to emerge in dorsal MEC. These different developmental timelines may be associated with the different functional properties of these two cortical input sets and may thus impact the emerging properties of neurons in MEC. PaS, PrS, and RSC, together with EC, are key elements in the head-direction system processing visual, vestibular, and spatial information during navigation (Taube et al., 1990; Kornienko et al., 2018; Simonnet and Fricker, 2018). During postnatal development, a rudimentary head direction system is present before P14 and head-direction cells already have adult-like properties by P16, before the emergence of adult-like grid cell in EC (Langston et al., 2010; Wills et al., 2010; Tan et al., 2015). Earlier *in vivo* experiments have indicated that PrS/PaS-MEC projections develop adult-like electrophysiological features around this time as well (Canto et al., 2019). In contrast, only rudimentary and less discrete spatial firing of grid cells have been observed in MEC from the beginning of the third postnatal week and the firing patterns gradually mature during the third and fourth postnatal weeks. This is paralleled by the development of the POR-MEC projections which also acquire adult-like features only from the beginning of the second week of postnatal development before developing over the third and fourth postnatal weeks. Hence, this suggests that the POR-MEC projection might be relevant for the maturation of functionally defined cell types that achieve adult-like features later than head direction cells, such as grid cells.

POR has been suggested to be part of the egocentric representational system in the brain, likely involved in assessing the presence or absence of changes in context (Burwell and Hafeman, 2003). The projection from POR to EC might be crucial for the transformation of egocentric representations into an allocentric framework, as observed in EC (Gofman et al., 2019; LaChance et al., 2019). Interestingly, egocentric spatial orientation emerges before allocentric spatial orientation during postnatal development (Contreras et al., 2019; Fernandez-Baizan et al., 2021). This suggests that the postnatal changes in the POR-MEC projection must underlay postnatal cognitive changes.

Lastly, our study shows that the POR-LEC projection develops a bit later than the POR-MEC projection, thus suggesting that complex object-place-context associations, that apparently are LEC-dependent (Vandrey et al., 2020), might also develop later. Although it should be kept in mind that there is a controversy about how early simple object-context association emerges (Mullally and Maguire, 2014), our results are in line with functional studies in humans and rats reporting that spatial navigation develops first and more complex association skills, such as items-space/context associations, develop gradually between childhood and adolescence (Edgin et al., 2014; León et al., 2014; Westbrook et al., 2014; Ramsaran et al., 2016a, b). Our study provides new data about the cellular substrate that underlies the cognitive functions of the PHR, though further studies on the effect of *in vivo* manipulations of POR on EC function are needed to substantiate the functional impact of POR projections during the development of EC.

## Synthesis

Reviewing Editor: Lynda Erskine, University of Aberdeen Decisions are customarily a result of the Reviewing Editor and the peer reviewers coming together and discussing their recommendations until a consensus is reached. When revisions are invited, a fact-based synthesis statement explaining their decision and outlining what is needed to prepare a revision will be listed below. The following reviewer(s) agreed to reveal their identity: Thomas J Wills. Note: If this manuscript was transferred from JNeurosci and a decision was made to accept the manuscript without peer review, a brief statement to this effect will instead be what is listed below.

Your manuscript has been reviewed by 2 experts in the field who are agreed that the work is an important advance and will provide an invaluable resource for those studying circuit development relevant to learning and memory. Overall, they found the manuscript to be well written and the experiments rigorous and analysed appropriately. Below is a synthesis of the reviewers’ comments outlining some suggestions for improving the manuscript prior to publication. All these points should be able to be addressed through text changes. Thank you for submitting your interesting work to eNeuro.

General comments from the reviewers:

This study represents the first description of the development of the projection pathway from the Post-rhinal to Entorhinal cortex. I find this to be an important advance in describing the development of the extended hippocampal formation in the rodent, which will provide a valuable resource for those studying the developmental cognitive neuroscience, and developmental circuit neuroscience of the hippocampal formation.

Overall I find the paper is very well written, and describes a very extensive dataset which has been collected with rigorous technique and analysed appropriately.

The authors provide a thorough and sophisticated dataset to investigate the neuronal development and connectivity from postrhinal cortex to entorhinal cortex at early postnatal development. Since the parahippocampal is crucial for learning and memory, it is important to fully understand the basic circuit mechanism. This study provides new insights of circuit mechanism in both anatomical and functional aspects during the early development, which enriched the understanding in the field.

Overall, authors used sophisticated tracing techniques and slice recording to show the developmental progress of POR to EC at early postnatal. The data are generally convincing with good sample size and proper statistics.

Points to address in a revised manuscript:

1. One of the main concerns of this manuscript is their categorization of the dPOR and vPOR. Although, the authors put a lot of efforts in providing comprehensive histology data and their flatmap analysis, some important aspects still need to be explained and addressed.

a) what is the BDA injection volume and coordinates for the animals. Did the authors purposely target dPOR vs vPOR, or did they just got it from variation of the injections? The authors should give a full, detailed explanation of which external landmarks and coordinates they used to target the POR, in addition to the volumes, so that these studies can be replicated.

b) the dPOR and vPOR from the representative images of Fig.6 A-D and 7 A-D show a large proportion of overlap in the middle part, which makes the categorization less powerful. In Fig.4, the authors show a good amount of flatmap data, which is really good. If the dorsal and ventral categorization is based on the flatmap, what is the criteria for that?

c) for Table 1, the authors try to preserve the first hand observation for the readers, which is really good. However, the information in there is a little bit confusing. What does "Number" mean? Does it stand for animal or slice number? Why did some animals get labeled in Superf. dPOR without Deeper dPOR could also have vPOR labeled. Does that mean the diffusion of the tracer is somehow not evenly in the brain.

d) do authors use the PV/CR staining as they showed in Fig.2 to confirm all their dorsal ventral categorization?

2. In line 471-472, the authors used k-mean cluster to analyze the pattern of MEC labeling and injection site. And they claimed they found 2 clusters associated with position. Full details of the k-means clustering should be provided, otherwise it is difficult to interpret the meaning of the two injection site groups created.

3. Line 86: The authors state that object-in-context memory emerges in middle- or late- childhood. This might be somewhat oversimplifying the field. There is some controversy over how early simple object-context associations can emerge, and if they do emerge early, whether they are supported by hippocampus or other areas. The field is helpfully reviewed by Mulally & Maguire, 2014, Dev Cogn Neurosci. The authors should make some reference to these open questions in the cognitive field.

4. Line 95: the authors state that "the firing fields of grid cells, though present around P16, evolve more gradually into adult-like functional neurons between P16 and P34" However, one of the cited studies, Wills et al 2010, found no evidence of grid cell firing at P16, followed by an abrupt emergence at P20. Possibly a form of words that encompasses the findings of both of the cited studies would be ’Immature grid cells in mEC show only disorganised spatial firing at P16, and adult-like grid cells emerge between P16 and P30.’

5. Line 542-543: The statement that ’The number of synapses also increased between the 543 second and the third postnatal week (figure 11 C).’ could have been better supported with a description of the methods used to count the synapses, and some illustrative raw data to support the population counts. The authors should add these, to bring this section up to the same standards as the rest of the manuscript.

6. For Fig.12 A-H, what are the timestamp for those images (how long post stimulation)? J-J’ could use better color coding. Adding some sample recording tracing in this figure will also make it easier to understand. For example, 8 parallel traces from one recording session, like what is shown in I.

7. Line 476-481: Testing for an age-region interaction effect in a 2-way ANOVA would demonstrate a development-specific change in POR projection targeting.

8. Sections of the text that need modified/clarified:

(i) Line 50: the word ‘essentially’ appears redundant in the abstract.

(ii) Line 85: ‘develops after birth’ is redundant here (cognitive memory not testable before birth?).

(iii) In line 343, "PER" is not explained anywhere in the text.

(iv) In line 369, "layer Va", could be a typo.

(v) In Methods, "Surgeries section, What is the injection volume for BDA.

(vi) In Methods, Surgeries section, author mentioned fast-blue or retrobead green were used for MEC retrograde tracing. However, in Fig.9, fluorogold (FG) was used in A and D, not retrobead green. Is this a typo in the figure legend. Moreover, can the authors explain why they used different tracers for different age rats?
